# DC traction grid modernization strategy to support EV chargers integration, enhance grid performance and profitability

**DOI:** 10.1038/s41598-026-53377-8

**Published:** 2026-06-04

**Authors:** Ievgen Verbytskyi, Mykola Lukianov, Mikołaj Bartłomiejczyk, Serhii Denysiuk, Ryszard Strzelecki

**Affiliations:** 1 Departament of Electronic Devices and Systems, National Technical University of Ukraine Igor Sikorsky Kyiv Polytechnic Institute, Prospect Beresteisky 37, Kyiv, 03056 Ukraine; 2 Department of Power Electronics and Electrical Machines, Gdańsk University of Technology, Gabriela Narutowicza 11/12, 80-222 Gdańsk, Poland; 3 Institute of Electrodynamics of the National Academy of Sciences of Ukraine, 56, Beresteyskyi Ave., Kyiv, 03057 Ukraine; 4 Faculty of Electrical Engineering, Gdynia Maritime University, 81-87 Morska St., Gdynia, Poland

**Keywords:** DC traction grids, EV chargers, RES, optimization, Energy and society, Energy science and technology, Engineering

## Abstract

This paper proposes a multi-stage modernization strategy based on return on investment (ROI) analysis for integrating EV chargers, battery storage and renewable energy sources (RES) into DC traction grids. A power converter interface (PCI), which consists of multiple power converters and a Smart Grid–based integration concept is proposed for modifying the existing infrastructure. The approach combines technical and economic considerations to support decision-making under varying regulatory and operational conditions. The effectiveness of the approach is evaluated using representative cost models, showing that additional services enabled by the PCI, such as EV charging, power quality improvement and RES integration, can achieve ROI values in the range of 10–20%, depending on electricity tariffs, penalty coefficients and utilization levels. The results indicate that, under suitable conditions, staged modernization of traction substations can improve power quality, reduce overloads and increase infrastructure utilization.

## Introduction

The development and integration of public electric transport in urban areas contributes to significant societal benefits, including reduced greenhouse gas emissions, lower noise pollution, improved air quality and, consequently, enhanced public health and overall urban living conditions^1^. In this context, grid-powered electric transport vehicles such as trams, trolleybuses and trains have long been valued for their comfort, operational simplicity and environmental friendliness^2^. At the same time, advancements in high-capacity energy storage technologies are driving the rapid growth of fully electric vehicles (EVs). In parallel, many developed countries are planning to phase out the production of internal combustion engine vehicles between 2035 and 2050^3,4^. While these developments are primarily motivated by environmental objectives, they also introduce potential technical challenges, including increased stress on electric power systems and potential risks to grid stability^5^. The risks are mainly associated with the growing reliance on renewable energy sources (RES) to ensure that EVs are charged with clean, low-emission electricity. The RES are inherently unstable and can increase energy system volatility. As a result, due to the phenomenon of simultaneous electric vehicle charging at peak hours^6^, the instability of the system additionally increases and may become critical. Consequently, modern research focuses on integrating EVs into modern electric power systems using Smart Grid concepts, Vehicle-to-Grid (V2G) charging^7,8^ and related technologies, in which aggregated EV battery storage systems serve as stabilizing elements for distributed power networks. It is unlikely that fully autonomous EVs will replace grid-connected traction transport for large-scale passenger and cargo applications in the coming decades. This implies that fully autonomous EVs and grid-powered electric transport will coexist, creating various opportunities for integrating EVs into traction grid infrastructure. Such integration can address the following tasks:

- improvement of traction grid power quality parameters;
- ensuring the function of uninterrupted power supply;
- distributed RES integration into traction grids;
- improving the schedule of energy consumption from traction substations;
- mitigation of the synchronization effect of electric car charging.

Despite extensive research on DC traction systems EV integration and renewable energy deployment, these domains are typically addressed independently. Existing studies often focus on component-level improvements (e.g., converter topologies or storage technologies) or specific functionalities such as V2G services or active filtering, without providing a unified framework for coordinated system-level modernization. As a result, there is a lack of methodologies that simultaneously consider technical interactions, infrastructure constraints and economic feasibility when integrating multiple functionalities into existing traction grids. In particular, current approaches do not explicitly address the problem of how to sequence modernization actions under real-world constraints, where investment decisions depend on regulatory conditions, penalty mechanisms and service utilization. This creates a gap between technically feasible solutions and economically viable implementation strategies. To address this limitation, this paper proposes a multi-stage modernization framework based on ROI analysis, supported by a power converter interface (PCI) that enables integration of EV charging, energy storage and RES within existing DC traction infrastructure. The main contributions of this work are:

- a system-level framework for coordinated integration of EVs, storage and RES into DC traction grids;
- a staged modernization roadmap, linking technical upgrades with economic feasibility conditions;
- an ROI-based decision model that identifies conditions under which specific functionalities become economically viable;
- a comparative analysis of converter topologies and system configurations from both technical and cost perspectives.

Unlike purely technical studies, the proposed approach focuses on decision support for infrastructure modernization, where simplified models are intentionally used to capture dominant effects while maintaining analytical tractability.

Chapter 2 in details analyzes DC traction grid optimization opportunities by reviewing traction grid issues and their potential solutions. Chapter 3 shows a structure of the proposed power converter interface, that allows integrating EV chargers, renewable energy sources, storage batteries and other appliances into DC traction grids. Chapter 4 analyses economic feasibility of power converter interface implementation. Chapter 5 proposes traction DC grid substation modernization stages for improved efficiency and profitability.

## DC traction grid optimization opportunities

### A. Traction DC power grids review and issues

**Fig. 1:**
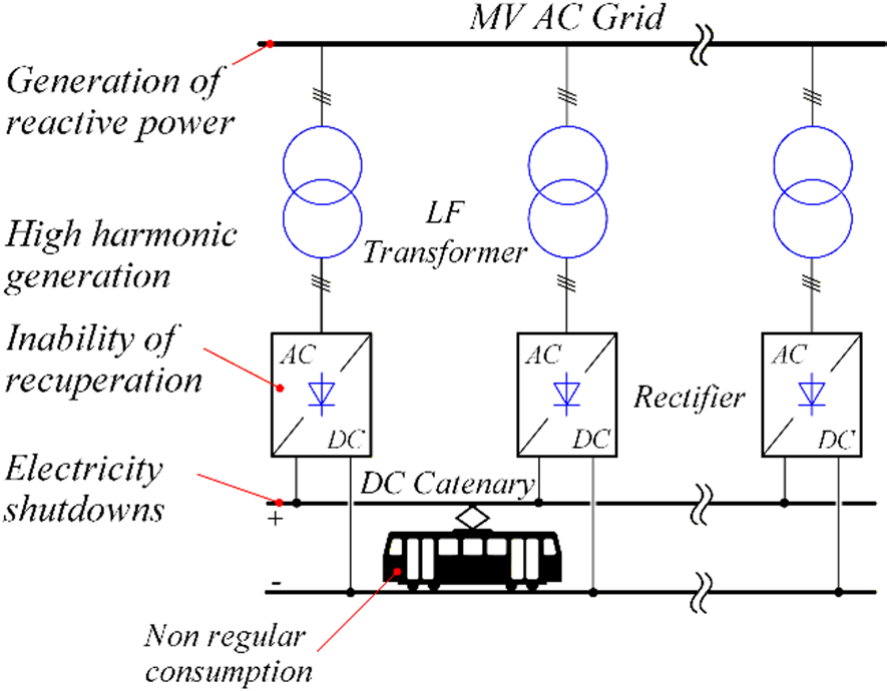
The typical structure of a traction DC grid with the associated problems being highlighted.

Nowadays, different types of DC traction grids are used: 750 V (600 V for older grids), 1.5 kV and 3 kV^9^. A 750 V grid is mainly used for urban trams, trolleybuses and light metro trains, whereas 1500–3000 V for suburban transport, metro and heavy metro. Basic structure of the traction DC grid consists of several substations, distributed at some distance from each other (Fig. 1), which include low frequency step down transformer and AC-DC converters (usually diode rectifiers). Such a configuration of traction grid leads to a number of issues (Table 1).

**Table 1:** Issues of DC traction grids with common configuration.

Issue	Reason	Impact
Generation of reactive power^10^	Electric lines reactance between rectifier and step-down transformer	Reactive power generation by substation
High harmonic generation^10^	Passive rectifier topology	High THD of substation input current, THD > 0.3
Inability of recuperation^11^	One direction energy flow	Decreasing efficiency of traction system, DC line overvoltage
Non regular consumption^12^	Pauses in the movement of transport during the day and no movement at night	Low effectiveness of substation equipment operation
Electricity shutdowns^13^	Lack of energy storage	Emergency stops of transport
Power supply from powerful sources of electricity, mainly based on fossil fuel^14^	Centralized power supply scheme	Environmental pollution

**Table 2:** Set of actions to mitigate problems of the traction power grids.

Level	Action	Effect
Component	The use of silicon carbide (SiC) and gallium nitride (GaN) semiconductors^15^	Reduced static and dynamic losses; higher switching frequency, thus, reduced weight; increased voltage, thus reduced power losses
	The use of transistors instead of diodes^16^	Reduced losses due to active rectification, bidirectional energy transfer
	Applying a high-frequency smart transformers instead of 50 / 60 Hz transformers^17^	Substations weight reduction
Unit	The use of multi-pulse rectifiers (12-, 18-, 24-pulse)^18^	Reduced amplitude and increased DC voltage ripple frequency, thus, reduced output filer size
	Installation of reactive power compensators, active filters, balancing devices, PFC devices^19^	Reduced THD and power losses in power lines
	Implementation of bidirectional converters topology and power-conditioning systems^22^	Energy recuperation, energy storage, distribution energy system organization, renewable energy sources integration
System	Smart grid^7^ (Storage system and renewable energy sources integration to traction grid, intellectual control and effective energy distribution)	Reduced maximum demand on a substation as well as transformer capacity reduction
	Vehicle to grid^8^	The method of attracting EVs for sustainable DC grids operation
	Smart metering^25^	Stimulation of EV owners to provide traction power grids balancing

Those issues force improving the topology of the power grid infrastructure and implementing new technologies and concepts, that are focused on sustainable development of the energy industry, based on renewable energy sources and distributed generation, as well as increasing system efficiency. For providing the aforementioned goals a number of multifaceted actions can be implemented, which are divided into different levels - component, unit and system levels (Table 2). The component level actions do not change the topology of transport infrastructure, but only replace existing components with more efficient ones. The unit level actions assume significant topology revision of the separate units and improving their effectiveness thanks to better organization of electrical energy conversion parameters. The system level involves rethinking the interaction of units, their purpose and functionality as a whole. At the same time, changes on the system level should be carried out together with the unit and component levels, which allows achieving maximum overall system efficiency.

The issues mentioned in Table 1 may be partially mitigated without significant changes at the system level. Thus, for electricity shutdowns issue, ring configuration can be used to increase the reliability of the power supply by duplicating equipment and power lines. To solve the issue of high harmonic generation, multi-pulse topology^26,27^(3-winding transformer, connection star-star, star-delta) can be used, which reduces THD, increase operating voltage and reduce the weight of the substation output filter. However, it does not solve the problem of traction transport braking energy recuperation and THD level is usually not sufficient. To solve the issue of THD and reactive power generation, thyristor reactive power compensators (STATCOMs^28^ are used, which allow compensating reactive power to high extend and provide high THD level. To achieve braking energy recuperation function, grid-synchronized thyristor inverters^29^ are used in parallel to the substation’s passive diode rectifier. However, this solution typically has low efficiency and high inertia, which can lead to line overvoltage.

As can be seen, abovementioned solutions have their benefits and drawbacks, different structure, number of components and price, which makes a choice of a specific solution for a DC grid modernization quite difficult. To solve the problem, this article presents a multi-stage traction grid modernization strategy, based on a return on investment (ROI) calculation for a system, which integrates renewable energy sources, has improved compatibility with the power grid, reduced power losses, improved flexibility and intelligence of transport infrastructure, as well as integrated EV charging stations. The aim is to comprehensively solve the problem, mentioned in Table 1, while getting as high as possible return on investment coefficient. To achieve the goal, paper describes mathematical methods to determine total system ROI, based on penalties and potential profit from the system modernization, as well as qualitatively compares different modern converter topologies, that potentially can be used for the system modernization (PV, EV converters and AC-DC grid inverters).

### B. EVs electrification trends and prospects of shared traction DC grids

According to the base forecast the share of electric cars in overall car sales is set to exceed 40% in 2030 under today’s policy settings^30^. However, such a rapid growth is associated with a problem, that EVs are an additional consumer of electricity and their maintenance involves an increase in electric generation as well as the infrastructure capacity, i.e. charging stations, power lines and substations. Simultaneously, according to Net Zero Emission Scenario^31^, electricity generation by 2050 has to increase approximately 2 times whereas share of electric generation of renewables (wind and solar) will reach 15% in 2030 and 28% in 2050^32^. As a result, to meet future demands, storage systems infrastructure for the RES must grow proactively. However, the required capacity can be reduced by smart use of existing power systems and integrating electric vehicle batteries as a distributed storage, as in the Vehicle-to-Grid (V2G) concept. Due to dense location of urban DC traction grids, where EVs are most common and dense substation locations, DC traction grids may serve as a point of common connection for RES and EV chargers. In such case, EVs can be charged near the owner’s residence, especially at night when public transport is inactive and substations are underused, helping balance the energy system. Daytime charging is also possible during transport downtime or energy recuperation from braking. At the same time, energy generated by RES is used locally, to power the traction transport as well for EV charging, reducing grid load and transportation losses.

Utilization of EV batteries in bidirectional mode eliminates substation power consumption peaks during the urban transport acceleration and may be used as a backup power supply. However, in this case, the car owner should have a more attractive tariff for charging the car, which covers the depreciation costs from the ageing of the battery. In general, a service company of the traction power grid has to organize a flexible tariff for car charging to balance energy consumption from the grid, which depends on modes that are analyzed in Table 3.

**Fig. 2:**
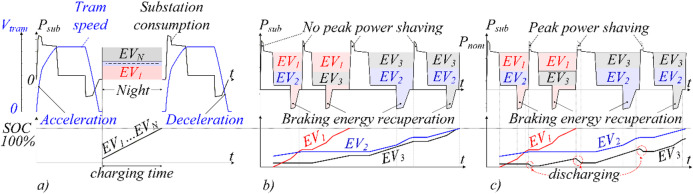
Illustration for modes of car charging: (**a**) night charging; (**b**) unidirectional daily charging; (**c**) bidirectional daily charging.

An illustration of charging modes analyzed in Table 3 is shown in Fig. 2. As shown in Fig. 2a at night all cars are charged identically and at the end of the night all cars are fully charged. The total charging power of all cars should be equal to the nominal power of substation *P*_*nom*_ which provide maximum operation efficiency and mitigates amount of reactive power. At daytime (Fig. 2b), cars are charged during intervals, when the substation does not feed urban transport or urban transport recuperates energy during braking. Higher charging priority, as well as larger tariff, have cars with shorter charging time as EV_1_ in Fig. 2b, c. In bidirectional mode, some car owners (Fig. 2c EV_3_) permit battery discharge to eliminate peak consumption from substation because of high energy consumption during traction transport acceleration, or other reasons.

As a result, integration of EVs to DC grids can play a balancing function for the traction system, but for their efficient integration, the whole system should be reviewed on the system, unit and component levels. Possible mechanisms of EV owner’s encouragement are explained in the next chapter.

**Table 3:** Modes of car charging from traction DC grid.

Mode	Sub mode	Features	Tariff	Penalties
Night (when the substation is idle)	Unidirectional	Car is charging all night permanently with constant current (power)	Lower than average	Not applied
Daily	Unidirectional	Car charging when the substation is idle and with recuperation, time of charging may be defined Charging time is defined by the user	From lower than average, when charging time is long, to larger than average when charging time is low Tariff depends on the time. During the maximum power demand (7–10 a.m., 5–8 p.m.) the tariff is larger	Applied for untimely end of car charging (earlier or later)
	Bidirectional	In addition to two unidirectional daily modes: User defines an amount of battery energy that may be used for grid balancing User defines a minimum battery state of charge at the end of charging (but as usual battery will be fully charged)	Lower than average with gradation depending on charging duration and time As larger amount of battery energy for balancing and lower and minimum battery state of charge at the end of charging as lower tariff as lower tariff Tariff may be negative (service company pays to car owner)	

## A proposed traction infrastructure modernization for municipal transport

**Fig. 3:**
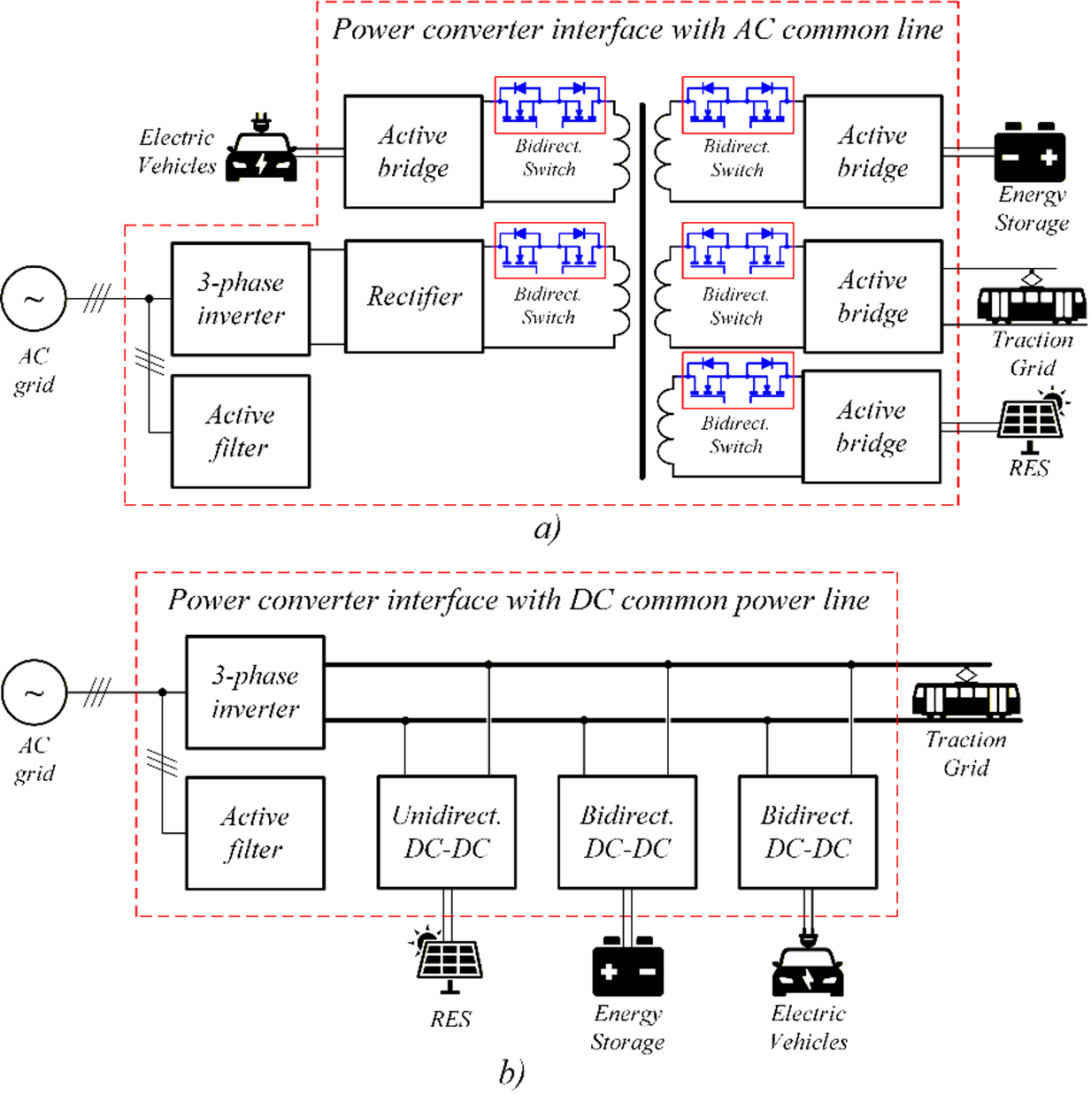
PCI based on distributed system with common AC (**a**) and DC (**b**) power line.

To modify municipal transport infrastructure, a power converter interface (PCI) is proposed, which presents a system that integrates AC grid, DC traction grid, electric vehicles, renewable energy sources and extra energy storage. The PCI can be either based on a common AC power line (Fig. 3a)^33^ or common DC power line (Fig. 3b)^34^. The multiport PCI (Fig. 3a) is mostly consists of active bridge converters and allows use transport and charging infrastructure with non-unified voltage levels, delivering energy between DC units with DC-AC-DC power conversion stage. However, to interface three phase grid, it still needs an additional power conversion stage, such as 3-phase inverter. Additionally, each unit contains bidirectional switch for the opportunity to exclude some unit in case of emergencies. The PCI with common DC power line (Fig. 3b), in its turn, uses DC traction grid as a point of common coupling and mostly uses transformerless converters (except EV connection), therefore it is cheaper and has higher efficiency. From the other side, due to the absence of the high frequency transformers, voltage matching between units is more complicated and requires more careful consideration of the converter topologies (Table 4).

To reduce modernization costs of the system infrastructure, it is considered that the substation remains untouched, with a unidirectional diode rectifier, that provides basic unidirectional energy flow from the AC grid. Although such configurations are known to exhibit limitations in terms of energy recuperation and harmonic performance, this assumption reflects practical constraints associated with legacy infrastructure, where full replacement of substations is often economically prohibitive. The proposed PCI is therefore designed as an incremental retrofit solution, enabling functionality extension without requiring fundamental changes to the existing supply architecture. This allows the analysis to focus on cost-effective modernization pathways, rather than idealized system redesign. Therefore, other units (PV, EV RES, reversible converter) are additional modification stages, aimed to improve system functionality (Table 5) if necessary and economically worth it. For example, extra stationary energy storage is usually required to store energy from renewable sources even when there are no EVs in the parking lot.

**Table 4:** Requirements for power units.

Unit	Isolation	Voltage range	Power converter features
Traction DC grid	Already isolated from AC grid	500–600 V Recuperation peaks up to 1000 V	None for DC power line; Dual active bridge for AC power line
Electric vehicles	Required	200–800 V (depends on EV battery type)	Wide range input/output voltage converter, most likely isolated
Energy storage	Optionally	Adjustable level *V*_*ES*_, voltage range *V*_*ES*_. 1.55*V*_*ES*_	Wide range input/output voltage converter, most likely isolated
RES	None	Adjustable level *V*_*RES*_, *voltage range* *V*_*RES*_. 1.4*V*_*RES*_ (for solar battery)	Non-isolated converter with MPPT feature
AC grid	Required	3-25 kV ± 10%	Isolated active filter

**Table 5:** Options for traction grid modernization.

Energy flow	Feature	Requirements
Return energy flow	DC-AC unidirectional energy flow	Power factor correction Active/ reactive power generation
DC power grid exchange	DC-DC bidirectional energy flow	Low latency high speed energy transfer link Power line protection from under/ overvoltage Provision of backup power supply from energy storage / EVs
Battery charge-discharge	DC-DC bidirectional energy flow	Charge energy storage with constant current (CC), constant voltage (CV) mode in traction grid idle mode Discharge energy storage (EVs) during peak stress of traction grid
EV charge-discharge		
Renewable energy source (RES) generation	DC-DC unidirectional energy flow	Maximum power point tracking

Certainly, it is possible to deliver this energy directly to the grid, but this increases energy transportation losses, reduces system stability and flexibility in covering energy shortages during peak load stress. Regardless of the connection structure and converter topologies used, PCI design has to provide efficient power exchange between power units with requirements mentioned in Table 4. As depicted in Table 4, DC-DC converters for EV charging stations have to have electric isolation, because both the instability of the traction DC grid voltage and the wide operation voltage range of batteries. The active filter also contains transformer because of different voltage levels of DC and AC traction grid.

It is worth noting that the literature also considers other power converter topologies for distributed power supply systems, which are typically based on combinations of AC and DC power lines^35^. Definitely, as a basis, the topology with common DC line has greater prospects, but final choice of one or another topology depends on a number of factors, among which it is possible to highlight electric isolation requirements, voltage range of input and output voltage of units as well as voltage of the traction grid.

## Power converter interface economic feasibility analysis

### A. Power factor penalties and harmonic distortions problem

**Fig. 4:**
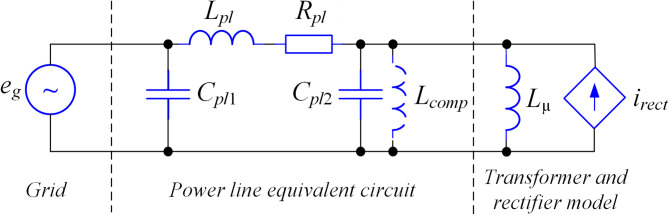
Equivalent electric circuit of the substation.

DC substation is connected with medium voltage power grid 3–25 kV via power cables. If power line is too long, its impedance creates significant level of reactive power, whereas passive input rectifier creates current distortions. Equivalent electric circuit of the substation is shown in Fig. 4, which consists of capacitors *C*_*pl*1_, *C*_*pl*2_, inductor *L*_*pl*_ and resistance *R*_*pl*_ that represent equivalent impedance of power line.

Transformer impact to the power flow is defined by magnetizing inductance *L*_µ_ that may be estimated by transformer cos(φ) value and controlled current source *i*_*rect*_ of rectifier input current. Total reactive power of such a line can be calculated as follows:

1 $${Q_\Sigma } = {Q_{pl}} - {Q_{tr}} = \omega {C_{eq}}U_g^2 - {S_{tr({\mathrm{nom}})}}\sqrt {1 - \cos {{(\varphi )}^2}},$$

where *Q*_*pl*_ is a power line reactive power, *Q*_*tr*_ – substation transformer reactive power, caused by magnetizing inductance *L*_µ_, that partially compensates power line reactive power; *ω* is a voltage angular frequency; *U*_*g*_ is a medium power grid RMS voltage; *C*_*eq*_ is a power line equivalent capacitance; *S*_*tr*(nom)_ is transformer complex power; *φ* is phase shift between first harmonics of grid voltage and current.

Reactive power *Q*_Σ_ is permanently present in the point of DC substation connection to the medium AC power grid, worsens power factor, especially with light load, causing penalties. For *Q*_Σ_ mitigation, compensating inductor *L*_*comp*_ may be added. Hence, total reactive power may be decreased to a level, that allows achieving required cos(φ) value when DC grid is loaded. However, in an idle mode, cos(φ) may still have unsatisfactory values. Substation input rectifier current *i*_*rect*_ does not worsen cos(φ) because its first harmonic is in phase with grid voltage but contains higher harmonics and, as result, worsens power factor PF.

Therefore, implementing PCI may improve operation of power grid in follows:

- average and redistribute the energy consumed from grid, which is especially important during the night;
- eliminate substation overload at peak hours;
- eliminate input current higher harmonics, while reactive power compensation is provided by passive circuits.

Supposing that nominal traction substation power is *P*_*TS*(nom)_, total reactive power generated by power lines after passive compensation is *Q*_Σ_, admissible value of cos(φ) is cos(φ_max_), cos(φ) ≥ cos(φ_max_) and power factor *PF* > *PF*_(min)_, minimum power *P*_*TS*(min)_ of the traction station with admissible value of cos(φ_max_) is calculated as:

2 $${P_{TS(\hbox{min} )}}={Q_\Sigma }\frac{{\cos ({\varphi _{\hbox{max} }})}}{{\sqrt {1 - \cos {{({\varphi _{\hbox{max} }})}^2}} }}P{F_{(\hbox{min} )}}\sqrt {1+{\mathrm{TH}}{{\mathrm{D}}_{TS}}^{2}} ,$$

where THD_*TS*_ is THD of input current of traction substation.

Obviously that restriction (2) is particularly sensitive for the night hours when public transport does not operate. Therefore, for penalties eliminating at night hours when traction substation operates in idle mode it is necessary to ensure the charging of electric vehicles with the total power *P*_*ch*_ which exceeds *P*_*TS*(min)_, *P*_*ch*_ ≥ *P*_*TS*(min)_. However, taking into account the variability of the demand for EV charging service, it is necessary to analyze additional possibilities for penalty eliminating with PCI when *P*_*ch*_ < *P*_*TS*(min)_, such as:

- additional power consumption in idle mode by additional energy storage;
- reactive power compensation or/and active power filtering with a recuperation power converter unit of PCI.

Usually, for the substation input energy flow, penalties on cos(φ) and power factor PF are applied, which reduce traction substation operation profit. In order to eliminate the penalties, using PCI, it is necessary to consider three possible connections: (1) EV chargers + energy storage, used when generated by EV power is not enough, or EVs are absent; (2) EV chargers + recuperation unit is used to improve current shape, compensate reactive energy as well as return power to the power grid; (3) EV chargers + both recuperation unit and energy storage are used for more flexible interaction with power grid. Appropriate solution for traction substation with EV charging depends on set of conditions: substation power *P*_*TS*(min)_, type of rectifier and penalty conditions, electricity tariffication, demand for charging service and so on. Hence, first of all, implementation of PCI based on proposed solutions should be analyzed and after that their effectiveness under certain conditions should be clarified.

Assuming that the traction substation configuration is defined with set of parameters:

- substation nominal power *P*_*TS*(nom)_;
- input current THD_*i*_ that defined with rectifier type;
- substation reactive power *Q*_Σ_.

Power quality requirements are defined with parameters:

- admissible value of cos(φ_max_);
- power factor *PF*_(min)_.

Electricity cost *C*_*e*_ and penalties for non-compliance with values of electricity quality *C*_*p*_ are the key factors determining the feasibility of moving to a new system level based on PCI. Therefore, the costs of transition to a new system level should pay off due to the reduction or complete elimination of the penalties and benefits from the provision of new services. Penalty fee is considered proportional to reactive power generation and difference between real and necessary power quality parameter, i.e.:

- for admissible value of cos(φ_max_) (if cos(φ) < cos(φ_max_)):

3 $${C_{\Sigma p}}={C_p}{Q_\Sigma }\int\limits_{0}^{T} {(\cos ({\varphi _{\hbox{max} }}) - \cos (\varphi (t)))} dt,$$

where *C*_*p*_ is cost for kVar·h or reactive power, *C*_Σ*p*_ is total penalty fee, *T* is observation period;

- for power factor (if *PF* < *PF*_min_):

4 $${C_{\Sigma p}}={C_p}{Q_\Sigma }\int\limits_{0}^{T} {(P{F_{(\hbox{min} )}} - PF(t))dt} .$$

According Table 6, EV chargers connection allows to reduce problems with penalties, but such option has non stable profit depending on EV number that is charged. Therefore, the additional stationary energy storage is usually required to reduce uncertainty and implement function of uninterruptable power supply. The active filter installing may additionally improve power factor and reactive power compensation. Such power system also may optionally integrate RES. As additional service such system may store energy at night and generate it in day hours if multi zone tariff is implemented.

**Table 6:** Benefits and penalties of different configurations of the traction substation.

Configuration	Penalties	Additional services
Basic	For admissible value of cos(φ_max_): ${C_{\Sigma p}}={Q_\Sigma }{C_p}\cos ({\varphi _{\hbox{max} }})T$	Not applied
	For power factor: ${C_{\Sigma p}}={Q_\Sigma }{C_p}P{F_{(\hbox{min} )}}T$	
EV charger + active filter + energy storage + RES (optionally)	For admissible value of cos(φ_max_):	EV charging Recuperation during breaking RES integration Feed-in tariff Input current distortion elimination Uninterrupted power supply Reactive power compensation Savings on the difference between day and night rates for electricity
	$${C_{\Sigma p}}={Q_\Sigma }{C_p} \times \int\limits_{0}^{T} {( {\cos ({\varphi _{\hbox{max} }})}}$$	
	$$- \frac{{{P_{ch}}(t)+{P_{ES}}(t)}}{{\sqrt {{{({P_{ch}}(t)+{P_{ES}}(t))}^2}+{{({Q_\Sigma } - {P_{af}})}^2}} } )} dt,$$	
	For power factor:	
	$${C_{\Sigma p}}={Q_\Sigma }{C_p}\int\limits_{0}^{T} {( {P{F_{(\hbox{min} )}} }}$$	
	$$- \frac{{{P_d} - {P_{af(d)}}{{}}} } {P_d} {\frac{{{P_{ch}}(t)+{P_{ES}}(t)}}{{\sqrt {( {1+{\mathrm{TH}}{{\mathrm{D}}_{TS}}^{2}} )( {{{({P_{ch}}(t)+{P_{ES}}(t))}^2}+{{({Q_\Sigma } - {P_{af}})}^2}})} }}} )dt$$	
	where *P*_*af*(*d*)_ is active filter power used for power distortion *P*_*d*_ elimination; *P*_*af*(cos)_ is active filter power used for reactive power elimination; in case some system component is not installed, its power in the formula equals to zero	

### B. Return of investment calculation

#### General conditions

The benefits of transition to a new system level on the basis of PCI can be expressed through an increase in the profit from operating the system or a reduction of penalties. Assuming that a transport company has a monthly income from the transportation of passengers *С*_*in*_ and in the cost structure of the expense items on electricity and penalties are *C*_*e*_ and *C*_*p*_ respectively. Then, the transition to a new system level by integrating PCI is profitable under the condition:

5 $${C_{in}} - {C_p} - {C_e}<{C_{in}} - {C_{p(m)}} - {C_{e(m)}}+{C_s} - {C_{PCI}}/{T_{lf}},$$

where *C*_*p*(*m*)_ is penalties of consumed electricity after PCI implementation; *C*_*e*(*m*)_ is cost of consumed electricity after PCI implementation; *C*_*s*_ is income from additional services provided by PCI; *C*_*PCI*_ is cost of PCI and its maintenance; *T*_*lf*_ is a PCI operation lifetime.

Among factors affecting the increase in profitability from the PCI operation, follows should be underlined:

1. Penalty level *C*_*p*_ is defined as a stimulating factor for transition to a new, more efficient technology.
2. A list of additional services that can be implemented after modernization:

- EVs charging allowing eliminating penalties for inadmissible electricity quality and efficient utilizing energy;
- multi-zone tariff allowing reduce the cost of charging electric cars and more effectively use a capacity of energy storage at night, also there is an opportunity to sell some part of energy at day, if active filter is applied;
- feed-in tariff that increases the expediency of generating energy into the power grid based on RES.

3. Capacity factor CF^36^, which can be expressed by the ratio of the generated power *W*_*g*_ within the observation period *T*_*obs*_ and product of installed power capacity of equipment *P*_*s*(*inst*)_ on observation period *T*_*obs*_:

6 $${\mathrm{CF}}=\frac{{{W_g}}}{{{P_{s(inst)}} \cdot {T_{obs}}}},$$

that defines cost of the equipment and, as result, return on investment (ROI) factor^37^. The goal of the system design is to maximize CF factor by a proper design of PCI topology on system level and following rational selection of unit circuit technology and components. Main ideas of the design are:

- multipurpose use of PCI units;
- involve PCI units to realization of additional services and, as result, increase system profit;
- reducing traction DC grid voltage deviation, for reducing installed capacity of PCI units.

#### Basic considerations

**Fig. 5:**
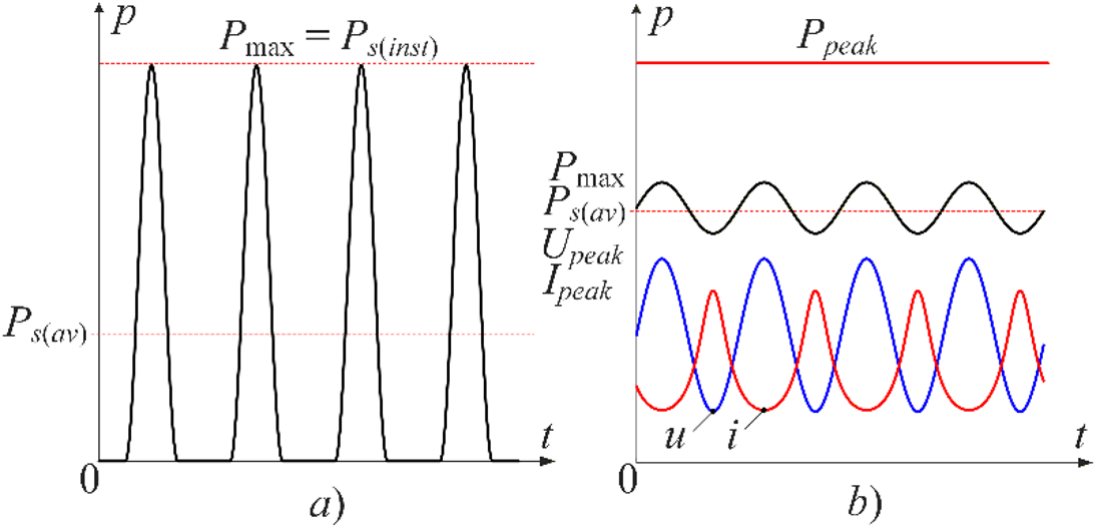
Illustration of power transportation problems: (**a**) unstable power flow; (**b**) unstable electric parameters.

The main reason for low capacity factor CF and, as result, low income, is a redundant installed equipment power capacity *P*_*s*(*inst*)_. Such problem is specific to systems with variable energy levels, e.g. power supply systems based on RES^38^, that require more than the market electricity tariff for their own payback (feed-in tariff). In such systems, power plant equipment is designed for a maximum power *P*_max_, that is equal to installed capacity *P*_*s*(*inst*)_, *P*_max_ = *P*_*s*(*inst*)_. However, usually it generates much less average power *P*_*s*(*av*)_, as shown in Fig. 5 (*a*). Such operation reduces the attractiveness of such energy sources and increases their payback period. Another problem that reduces capacity factor is the instability of the supply voltage, even if the power flow is almost constant. Such a problem is observed in traction grids, especially in recuperation mode, when overvoltage may reach 100% of nominal voltage^39^. Such instability forces to design substation equipment (transistors, transformers etc.) using components with much higher peak current *I*_*peak*_ and voltage *U*_*peak*_ values, resulting in a total systems power capability of *P*_*peak*_ = *I*_*peak*_ ·*U*_*peak*_, even though maximal power during its operation does not exceed *P*_*max*_ (Fig. 5b).

Therefore, the primary tasks of PCI design are reducing the instability of the transported power between power units and stabilizing the parameters of electrical energy, especially voltage of traction DC grid as the system-forming parameter. Economic benefit from PCI usage is estimated based on a common traction DC substation with nominal power *P*_*TS*(nom)_ and output nominal voltage *U*_*TS*(nom)_. As usual in such system voltage deviation is determined by input AC grid and does not exceed ± 10%, therefore minimum traction station voltage is *U*_*TS*(min)_ = 0.9*U*_*TS*(nom)_. Voltage spikes during traction transport recuperation, especially when there is no other vehicle that can consume the generated energy may exceeds 100%, *U*_*TS*(*peak*)_ > 2 *U*_*TS*(nom)_ and their duration is provided by Standard EN 50,163^40^ and in more details by Standard EN 50124-2^41^. Overvoltage of 70% with duration longer than period of AC grid, i.e. 20 ms, results in substation peak output voltage being *U*_*TS*(*peak*)_ = *U*_*TS*(max)_ = 1.7*U*_*TS*(nom)_.

As a result, the substation cost *C*_*sb*_ is defined based on product of maximum operation voltage and current:

7 $${C_{sb}}={k_p}{k_g}{U_{TS(\hbox{max} )}}{I_{TS(\hbox{max} )}}={k_p}{k_g}1.7{U_{TS(nom)}}1.1{I_{TS(nom)}} \approx 1.9{k_p}{k_g}{P_{TS(nom)}},$$

where *I*_*TS*(max)_ is maximum rms value of substation output current, *I*_*TS*(max)_ = *P*_*TS*(nom)_/ *U*_*TS*(min)_ ≈ 1.1 *I*_*TS*(nom)_; *k*_*p*_ is coefficient of proportionality, calculated later in the article; *k*_*g*_ is proportionality cost coefficient that depends of semiconductor devices type (for diodes *k*_*g*_ = 1 and for transistors *k*_*g*_ = 2), which shows the cost difference between power converters based only on diodes and modern transistor converters^42^. As result, typical substation is designed for power levels, which are 1.9 times higher than nominal power consumption, which proportionally increases cost.

Assuming transport route fed by *M* identical substations, which operate with overall lifetime income *C*_*in_lf*_ and route maintenance costs *C*_*a*_, electricity costs *C*_*e*_, penalties *C*_*p*_ and other operational fees. The overall profit *C*_*pr_lf*_ of the route during lifetime calculated as follows:

8 $$\begin{gathered} C_{pr\_lf} = C_{in\_lf} - M C_{sb} - T_{lf} MC_{a} - \\ T_{lf} MC_{e} P_{TS(nom)} {\mathrm{CF}} - T_{lf} Q_{\Sigma } C_{p} (\cos (\varphi_{\max } ) - \cos (\varphi )) - C_{oth\_in} - T_{lf} C_{oth\_cur} , \\ \end{gathered}$$

where *T*_*lf*_ is lifetime operation period; *Q*_Σ_ is reactive power generated by the substation; *C*_*oth_in*_ are other fixed fees, for instance price of transport EVs, cables, etc.; *C*_*oth_cur*_ are other operational fees, i.e. salary and equipment depreciation costs. The structure of income and expenses described in formula (8) is valid for the basic service of the transport route, namely the transportation of passengers. For simplification of subsequent analysis, normalized values of income and fees are used, defined for the section, powered by one substation, when income and fees are divided by *M*. Thus, the average annual ROI of route operation during one substation lifetime is:

9 $${\mathrm{ROI}} = \frac{{{C_{pr\_lf(1)}} - ({C_{sb}} + {C_{oth\_in(1)}} + {T_{lf}}({C_a} + {C_e}{P_{TS(nom)}}{\mathrm{CF}} + {Q_\Sigma }{C_p}(\cos ({\varphi _{\max }}) - \cos (\varphi )) + {C_{oth\_cur(1)}})))}}{{{T_{lf}}({C_{sb}} + {C_{oth\_in(1)}} + {T_{lf}}({C_a} + {C_e}{P_{TS(nom)}}{\mathrm{CF}} + {Q_\Sigma }{C_p}(\cos ({\varphi _{\max }}) - \cos (\varphi )) + {C_{oth\_cur(1)}}))}} \cdot 100\% .$$

Normalization is provided with common annual profit of municipal transport without penalties, which is typically ~ 10%^43^, corresponding to ROI = 10%. The share of each cost item is considered equal to: *shr* = 20%, i.e. shares of substation cost, other initial fees, depreciation substation cost, electricity as well as other current fees are the same and equal to 20%. Assuming a lifetime cycle for equipment *T*_*lf*_ = 15 years^44^, nominal power of substation *P*_*TS*(*nom*)_ = 1 MW and normalized lifetime profit *C*_*pr_lf*(1)_^*^ = 1.0 p.u., normalized initial substation cost *C*_sb_^*^ and other initial costs *C*_*oth*_*in*(1)_^*^ are equal to:

10 $$C_{sb}^{*} = C_{oth\_in(1)}^{*} = \frac{{shr \cdot C_{pr\_lf(1)}^{*} }}{{{\text{1 + ROI}} \cdot {\mathrm{T}}_{lf} }} = \frac{2}{25}{\text{ p}}{\mathrm{.u}}{.,}$$

whereas permanent fees for maintenance, electricity, etc. is equal:

11 $$C_{a}^{*} = C_{oth\_cur(1)}^{*} =C_{e}^{*} P_{TS(nom)} {\mathrm{CF}} = \frac{{shr \cdot C_{pr\_lf(1)}^{*} }}{{{\text{(1 + ROI}} \cdot {\mathrm{T}}_{lf} ){\mathrm{T}}_{lf} }} = \frac{2}{375}{\text{ p}}{\mathrm{.u}}{\mathrm{./year}}{.}$$

Comparing values *C*_*sb*_ (7) and *C*_sb_^*^ (10) and taking into account *P*_*TS*(nom)_ = 1 MW and *k*_*g*_ = 2, coefficient of proportionality *k*_*p*_ may be derived as *k*_*p*_ = 2·10^− 6^/95.

## Traction DC grid substation modernization stages

### Base scenario (no additional equipment is installed)

**Fig. 6:**
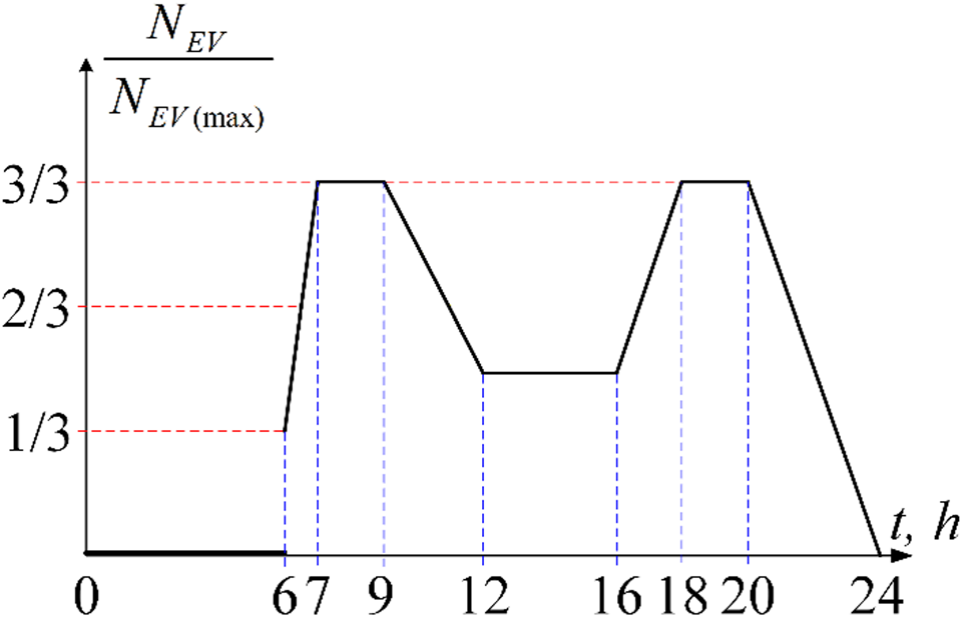
A normalized typical curve of the relation *N*_*EV*_/ *N*_*EV*(max)_ over time.

First of all, to calculate capacity factor CF for a typical traction substation, a maximum number of traction EVs *N*_*EV*_ (trams, trolleybuses) fed by the substation has to be defined. Taking into account the substation nominal power *P*_*TS*(*nom*)_ = 1 MW, typical nominal power of urban EVs *P*_*EV*(*nom*)_ = 200 kW and their peak power *P*_*EV*(*peak*)_ = 400 kW^45^, maximum number of EVs may be defined as *N*_*EV*(max)_ = 3. As shown in^46^, EVs consume energy in traction mode approximately 50% of total operation time *T*_*op*_. Thus, *T*_*Tr*_ = 0.5 *T*_*op*_ whereas recuperating duration *T*_*Rec*_ is 20%, *T*_*Rec*_ = 0.2 *T*_*op*_, and other time EV does not interact with power system, being stationary or moving by inertia. In the same time, even in traction mode, EV average power significantly less than nominal. Average to nominal EV ratio can be expressed as *P*_*EV*(*av*)_ / *P*_*EV*(*nom*)_ ≈ 0.2, which gives *P*_*EV*(*av*)_ =40 kW. Then, for EV nominal power *P*_*EV*(*nom*)_ = 200 kW, average consumed power in traction mode *P*_*EV*(*Tr*)_ = *P*_*EV*(*av*)_ · *T*_*op*_ / *T*_*Tr*_ = 80 kW. Substation utilization is even less, because substation is designed to operate with peak values, when maximum number of EVs *N*_*EV(max)*_ consume maximum power *P*_*EV(peak)*_, which during most of the day is not true. As a result, capacity factor for the traction substation with theoretical load profile (Fig. 6) is calculated as follows:

12 $$CF=\frac{1}{T}\int\limits_{0}^{T} {{N_{EV}}\frac{{{P_{EV(av)}}{P_{EV(peak)}}}}{{{P_{TS(peak)}}{P_{EV(nom)}}}}dt} .$$

It is clear that, the maximum number of EVs *N*_*EV*(max)_ on the line, powered by substation, operates only during peak hours. During other time a flexible schedule of EVs moving is applied. For simplifying penalties calculation, the power consumption *P*_*EV*(*Tr*)_ is assumed to be constant. Therefore, with number of EVs *N*_*EV*_ in range from 0 to 3, different consumption power levels are available: 0, *P*_*EV*(*Tr*)_, 2*P*_*EV*(*Tr*)_, 3*P*_*EV*(*Tr*)_. A total normalized duration of each power level.

may be defined via probability distribution for discrete random variables^47^, when mode of each EVs is considered as independent variable and probability of mode equals to *p*_*TR*_ =0.5.

**Fig. 7:**
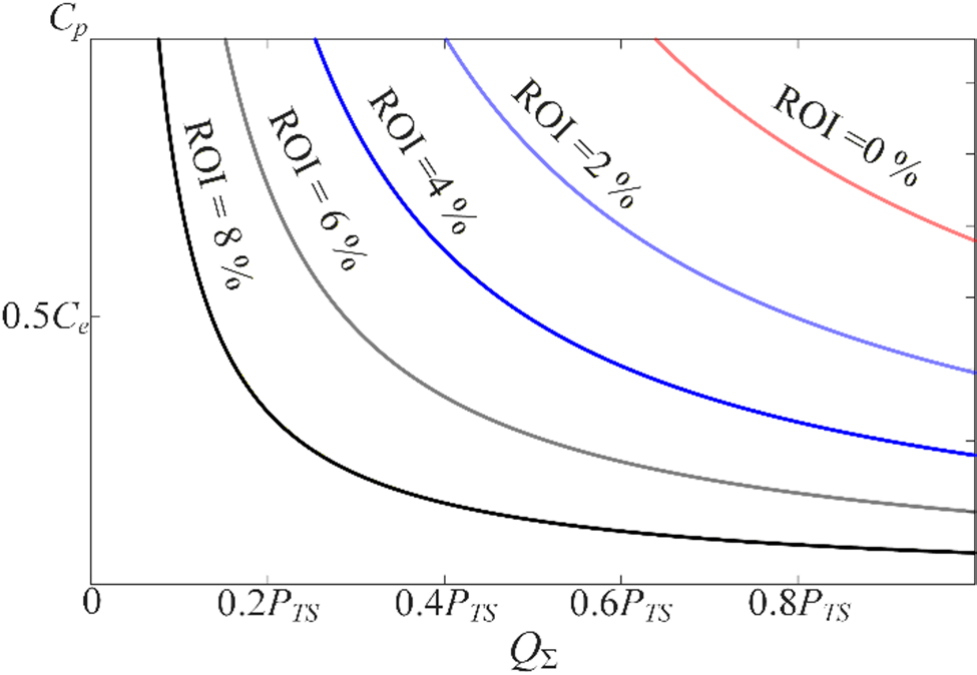
Variation of ROI depend on penalties and reactive power generation *Q*_Σ_ and penalties *C*_*p*_ for it.

For analyzed case with peak number of power trains powered from one substation being *N*_*EV*(max)_ = 3, *P*_*EV*(*av*)_ / *P*_*EV*(*nom*)_ ≈ 0.2, *k*_*peak*_ = 1.3, capacity factor CF after averaging curve of Fig. 6 is equal to CF = 0.08, which means that most of the day traction substation equipment is underused. The ROI in such case varies in wide range as shown in Fig. 7, depending on reactive power generation *Q*_Σ_ ∈ (0, *P*_*TS*_] and penalties for it *C*_*p*_ ∈ (0, *C*_*e*_] (considering penalties caused by *cos(φ*_*max*_*)* = 0.95 restrictions.) As a result, penalties may significantly decrease ROI and force to invest in new equipment to reduce them.

**Fig. 8:**
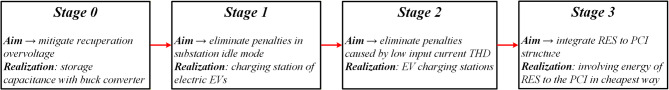
modernization roadmap with four stages of the power substation.

Type of new equipment and its profitability depends on penalty level, therefore, usually the system is being modernized sequentially with a gradual increase of penalties. The condition for each subsequent stage of modernization is to increase the profitability of the system after its modernization. Therefore, this chapter proposes traction DC grid modernization roadmap with four stages (Fig. 8). It is clear that a condition for moving to a new stage is increasing ROI after its implementation.

### Modernization stage 0

**Fig. 9:**
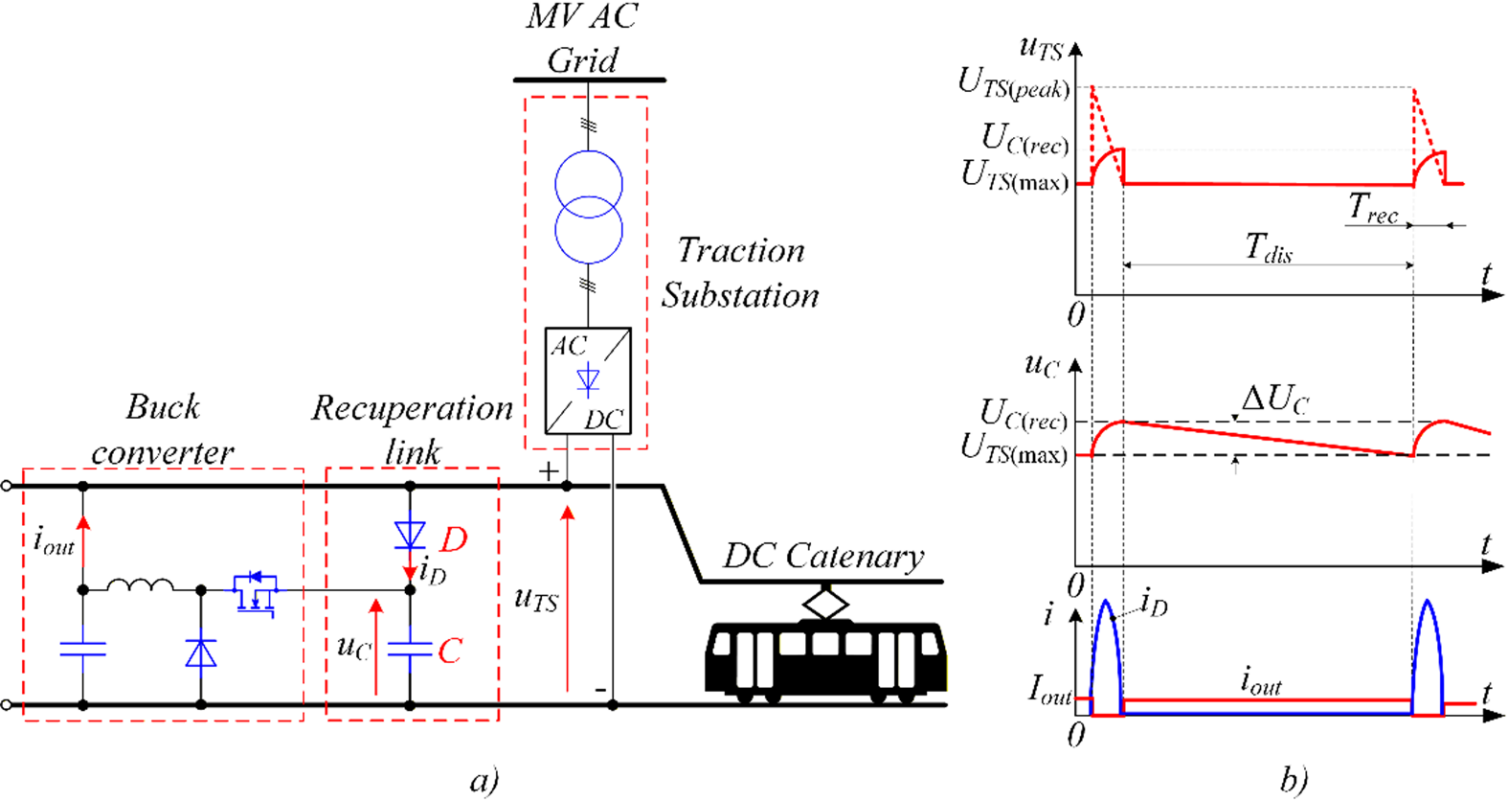
Link for overvoltage mitigation: (**a**) topology; (**b**) timing diagrams.

Modernization stage 0 is the preparatory stage, which is aimed at reducing overvoltage spikes and correspondingly the cost of the equipment that will be installed at the later stages. For overvoltage elimination a simple link with ultrafast diode *D*, capacitor storage *C* and buck converter as shown in Fig. 9 is proposed. Advantage of such a solution is a small installed capacity and, as result, its low cost.

It is clear that peak overcurrent on recuperation interval *T*_*rec*_ is achieved only in diode *D*. Whereas other components of the link is calculated on averaged, rather on peak current. That average current flow in buck converter during discharge period *T*_*dis*_ and returns in power line with current *i*_*out*_. Capacitance *C* is calculated based on recuperated energy *W*_*rec*_ and admissible voltage increase on the capacitor Δ*U*_*C*_. According to previous studies the recuperated energy *W*_*rec*_ is in range of 10% to 20% of the consumed energy *W*_*con*_, thus, *W*_*rec*_ = 0.1.0.2*W*_*con*_^48^. Therefore, capacitance *C* is calculated as follows:

13 $$C=\frac{{2{W_{rec}}}}{{{U_{C(rec)}}^{2} - {U_{TS(\hbox{max} )}}^{2}}},$$

where *U*_*C*(*rec*)_ is voltage on capacitor *C* immediately after recuperation.

Value *U*_*C*(*rec*)_ has to exceed value *U*_*TS*(max)_, therefore it defined as *U*_*C*(*rec*)_ = 1.2 *U*_*TS*(*nom*)_. Cost *C*_*st*0_ of the modernization stage 0 is mostly defined by most expensive component, i.e. transistor, and calculated as a product of its maximum current *I*_*Tr*(max)_ and voltage *U*_*Tr*(max)_:

14 $${C_{st0}}={k_g}{k_p}{I_{Tr(\hbox{max} )}}{U_{Tr(\hbox{max} )}},$$

where generation factor *k*_*g*_ = 2.

Transistor maximum voltage is the same as on capacitor *C*, *U*_*Tr*(max)_ = 1.2 *U*_*TS*(*nom*)_, whereas transistor maximum current *I*_*Tr*(max)_ is calculated based on recuperated energy:

15 $${I_{Tr(\hbox{max} )}}=\frac{{{W_{rec}}}}{{{T_{dis}}{U_{TS(\hbox{min} )}}}} \approx \frac{{0.15{W_{dis}}}}{{0.9{T_{dis}}{U_{TS(nom)}}}} \approx \frac{{{I_{TS(nom)}}}}{{30}}.$$

As result cost of the proposed link is *C*_*st*0_ = 0.08 *k*_*p*_*P*_*TS*(*nom*)_. Meanwhile, such link allows to reduce cost of substation equipment from 1.9 *k*_*p*_*P*_*TS*(*nom*)_ to 1.3 *k*_*p*_*P*_*TS*(*nom*)_ and additional equipment, installed later on. So, economy after initial modernization stage is 0.52 *k*_*p*_*P*_*TS*(*nom*)_ or 27% of the substation cost *C*_sb_^*^ and its maintenance *C*_a_^*^. Substitution of the new values in (9) gives a maximum ROI_max_ = 12%, so the improvement gives at least 2% extra profit annually.

### Modernization stage 1

The stage provides penalties mitigation during traction substation idle operation, as well as reduces grid power stress during EVs charging, utilizing the grid during substation idle state. In general, idle mode continuously occurs at night time from 11 p.m. to 7 a.m. that forces to organize a new service of EVs charging that provide such advantages:

- provide an additional profit *C*_*pr_lf*(*ch*)_^*^ from charging service;
- mitigate penalties;
- increase capacity factor CF of the traction substation;
- helps realize uninterrupted power supply and decrease overload of the substation.

**Fig. 10:**
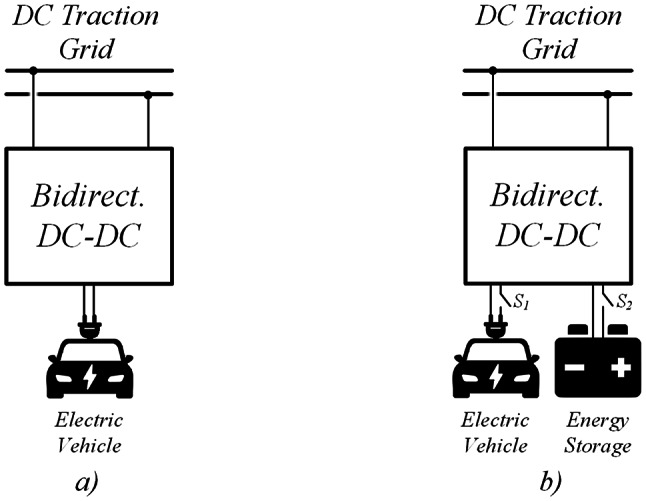
Charging solutions: (**a**) without extra battery; (**b**) with battery.

The main idea of the modernization is to load the substation with minimum active power *P*_*TS*(min)_ that eliminates penalties for generated reactive power *Q*_Σ_, defined with formulas (3) and (4). By analyzing profitability of the charging service *C*_*pr_lf*(*ch*)_^*^ independently, the ROI_*ch*_ of the charging service may be defined as follows:

16 $${\mathrm{ROI}}_{ch} = \frac{{k_{c} C_{pr\_lf(ch)}^{*} - (C_{ch}^{*} + T_{lf} (C_{a(ch)}^{*} + k_{c} (C_{e}^{*} P_{TS(nom)} {\mathrm{CF}}_{ch} - Q_{\Sigma } C_{p}^{*} (\cos (\varphi_{\max } ) - \cos (\varphi )))))}}{{T_{lf} (C_{ch}^{*} + T_{lf} (C_{a(ch)}^{*} + k_{c} (C_{e}^{*} P_{TS(nom)} {\mathrm{CF}}_{ch} - Q_{\Sigma } C_{p}^{*} (\cos (\varphi_{\max } ) - \cos (\varphi )))))}} \cdot 100\% ,$$

where *k*_*c*_ is level of the EV charging service use, *k*_*c*_ ∈ [0,1], *C*_ch_^*^ is cost of charging equipment, *C*_*a*(*ch*)_^*^ is cost for charging equipment maintenance, CF_*ch*_ is additional capacity factor of the substation created by the charging of EVs:

17 $${\mathrm{C}}{{\mathrm{F}}_{ch}}=\frac{{{p_{Id}}{P_{TS(\hbox{min} )}}}}{{{P_{TS(nom)}}}}.$$

Profitability of the charging service *C*_*pr_lf*(*ch*)_^*^ depends on difference between the charging service cost *C*_*s*(*ch*)_^*^ and electricity cost *C*_e_^*^. As usual charging service cost is 2–5 times higher than electricity cost^49^, *C*_*s*(*ch*)_^*^ = *k*_*s*(*ch*)_*C*_e_^*^, then potential profitability *C*_*pr_lf*(*ch*)_^*^ may be defined as:

18 $${{\mathrm{C}}_{pr\_lf(ch)}}^{*}={k_{s(ch)}}C_{e}^{*}{T_{lf}}{\mathrm{C}}{{\mathrm{F}}_{ch}}{P_{TS(nom)}},$$

that in practice is lowered with level of the EV charging service use *k*_*c*_.

As shown in (16), penalty costs are included as income due to their eliminating via the charging service. To get the maximum benefit from this stage of modernization, it is advisable to use a multi-zone electricity tariff, which provides reduced prices for electricity consumed at night. Then, electricity average cost *C*_*e*(*av*)_ will be a weighted parameter:

19 $${{\mathrm{C}}_{e(av)}}^{*}={{\mathrm{C}}_e}^{*}{k_d}+{{\mathrm{C}}_{e(n)}}^{*}(1 - {k_d}),$$

where *C*_*e*(*n*)_ is night electricity tariffs, *k*_*d*_ is share of electricity consumed during the day.

The condition for carrying out this modernization is higher ROI_*ch*_ of charging service comparatively to the transport service ROI, ROI_*ch*_ > ROI.

There are few options of EV charger’s installation. The simplest one is to install *N* standard bidirectional charging stations. But profitability of such solution very sensitive to charging service price for individual users. In general, to ensure that the charging station is filled with the required amount of EVs, the charging price offer should be lower than of other charging station in the proximity. This makes profitability of the penalty reduction service lower. Also, in order to implement the function of uninterrupted power supply and reduce the load on the substation, the permission of the EV user to discharge the battery is required. This necessitates additional charging price decrease to motivate EV users to give such a permission. The installation of an additional stationary battery storage, which can perform the same functions as EV batteries, can provide greater certainty in the operation of the system, but with additional cost. If multi-zone tariffication is applied it also leads to a new service based on difference between day and night tariffs and different ROI_(*ch_b*)_:

20 $${\mathrm{ROI}}_{ch} = \frac{{k_{c} C_{pr\_lf(ch\_b)}^{*} + k_{c1} C_{pr\_lf(el)}^{*} - (C_{ch}^{*} +C_{bat}^{*} + T_{lf} (C_{a(ch)}^{*} +C_{a(bat)}^{*} + \left( {k_{c} + k_{c1} } \right)(C_{e}^{*} P_{TS(nom)} {\mathrm{CF}}_{ch} - Q_{\Sigma } C_{p}^{*} (\cos (\varphi_{\max } ) - \cos (\varphi )))))}}{{T_{lf} (C_{ch}^{*} +C_{bat}^{*} + T_{lf} (C_{a(ch)}^{*} +C_{a(bat)}^{*} + \left( {k_{c} + k_{c1} } \right)(C_{e}^{*} P_{TS(nom)} {\mathrm{CF}}_{ch} - Q_{\Sigma } C_{p}^{*} (\cos (\varphi_{\max } ) - \cos (\varphi )))))}} \cdot 100\% ,$$

where *C*_*pr_lf*(*el*)_ is overall potential profit from service of charging/recharging battery with difference in day/night tariff, *k*_*c*1_ is level of the battery charging service use, *C*_bat_^*^ is cost of the set electric batteries, *C*_*a*(*bat*)_^*^ is cost for the battery maintenance.

**Fig. 11:**
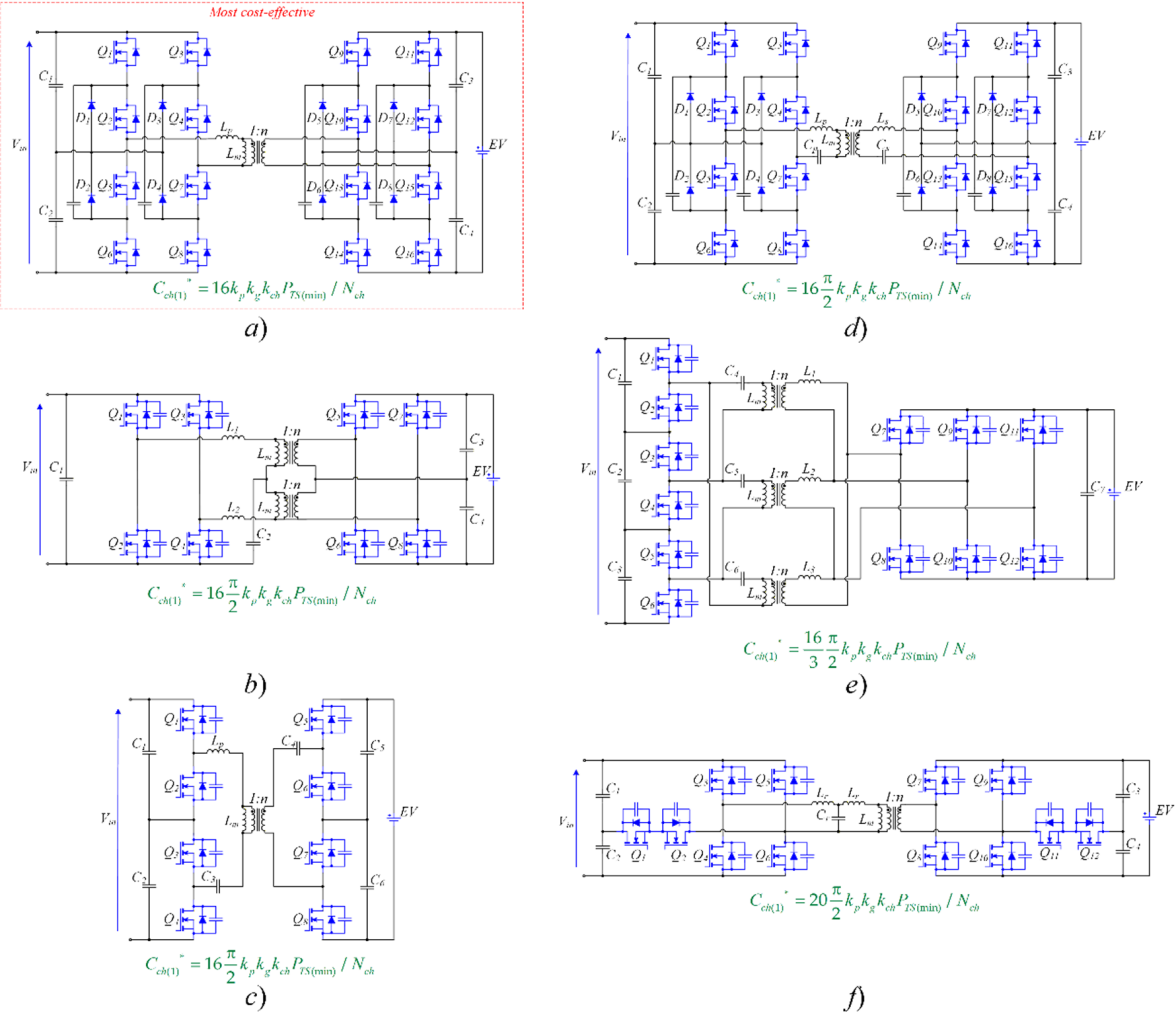
Single-stage charger topologies: (**a**) Neutral point clamped (NPC) DAB; (**b**) Reconfigurable DAB; (**c**) Reconfigurable LLC converter; (**d**) CLLC NPC DAB; (**e**) Three-phase Triple-Voltage LLC (T2-LLC); (**f**) Reconfigurable T-type DAB.

The cost of set electric batteries *C*_bat_^*^ may be defined via their total capacity *Cp*_*bat*_ and maximum operation voltage *U*_*bat*(max)_:

21 $${C_{bat}}^{*}={k_p}C{p_{bat}}{U_{bat(\hbox{max} )}}{\mathrm{.}}$$

The capacity *Cp*_*bat*(1)_ of the single battery within assumption of charging station of Level 2^50^ with medium charging speed 0.2–0.3 *Cp*_*bat*(1)_ has to provide the power consumption *P*_*TS*(min)_/*N*_*ch*_ during all idle intervals:

22 $$C{p_{bat(1)}}=\frac{{{P_{ST(\hbox{min} )}}}}{{0.2{N_{ch}}{U_{bat(\hbox{max} )}}}}{\mathrm{.}}$$

The charging service with an installed battery, except more stable profit and reliable elimination of penalties, is more efficient in eliminating the substation overload during EVs acceleration and reducing the power loss. Charging solutions with/without extra battery are shown in Fig. 10. A core of the proposed charging applications is bidirectional charger with wide voltage range. The charger cost is depended on traction station DC gird Δ*U*_*TS*_ and battery Δ*U*_*bat*_ voltage range, and on the charger converter topology.

The transistor cost is proportional to width of voltage operation range *k*_*ch*_:

23 $${k_{ch}}=\left( {\frac{{\Delta {U_{bat}}}}{{{U_{bat(\hbox{min} )}}}}} \right){\mathrm{=4}}{\mathrm{.}}$$

Another component of the single charger cost is defined by total transistor cost of the charger topology when charger operates with unity gain *G* = 1:

24 $${C_{ch(1)}}^{*}(G=1)={k_p}{k_g}\sum\limits_{{j=1}}^{N} {{U_{{Q_{j(\hbox{max} )}}}}{I_{{Q_{j(\hbox{max} )}}}}} ,$$

where *U*_*Qj*(max)_, *I*_*Qj*(max)_ are maximum voltage and current of transistor *Q*_*j*_ respectively.

**Fig. 12:**
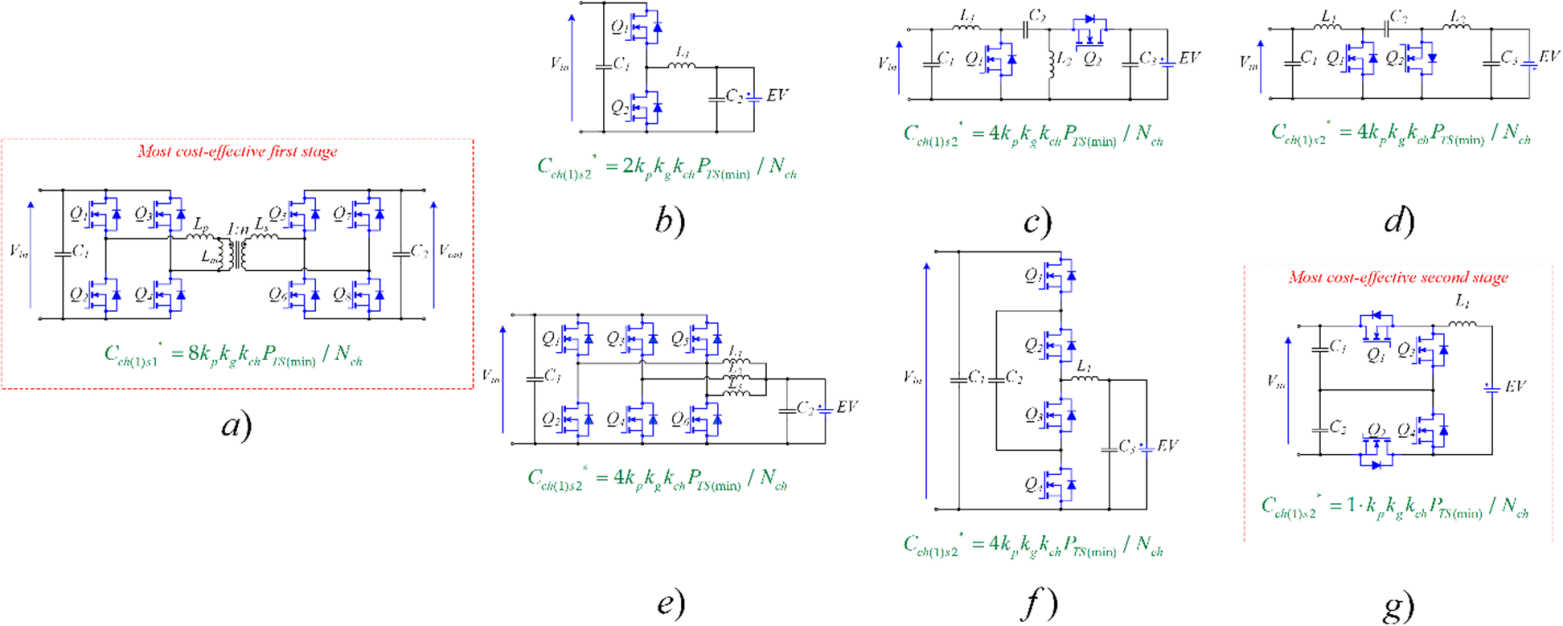
Stages of two-stage charger topologies: (**a**) first stage based on DAB; (**b**) second stage half-bridge; (**c**) second stage Cuk; (**d**) second stage SEPIC /Luo; (**e**) second stage interleaved half-bridge; (**f**) three level flying capacitor; (**g**) parallel three level buck.

Hence, for predefined voltage range that characterized by total charger gain *G*_*ch*_ its cost *C*_ch_^*^ is calculated as follows:

25 $${C_{ch(1)}}^{*}({G_{ch}})={k_{ch}}{C_{ch(1)}}^{*}(G=1).$$

**Fig. 13:**
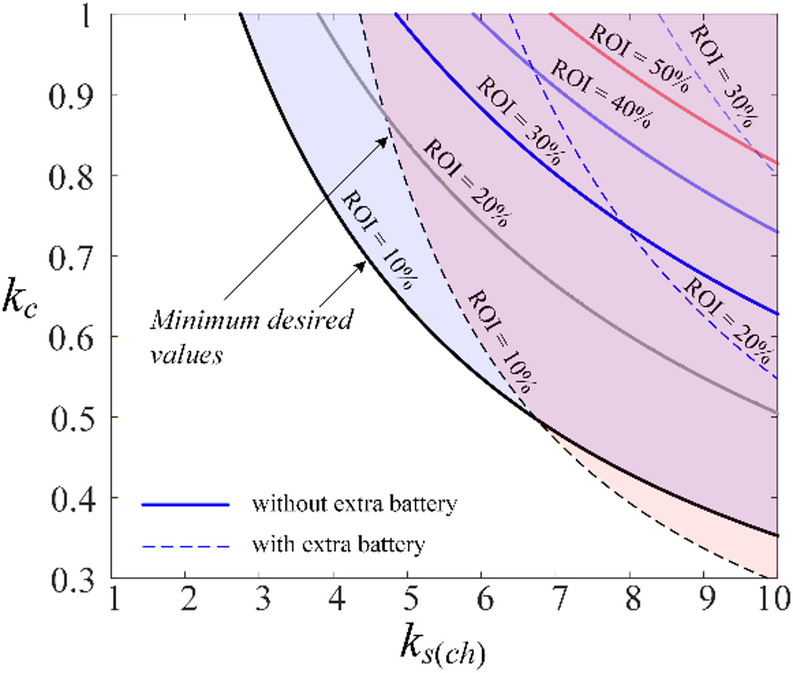
ROI_ch_ curves for both types of charging stations, with and without extra battery installed.

A common type bidirectional chargers are based on double active bridge (DAB) topologies^51^ but for wide voltage they have insufficient efficiency. For extension voltage range reconfigurable or multilevel converters based on DAB with isolation may be used. Reconfigurable converters due to extra switches or natural features embodies several converter topologies that allows it to operates in a wider voltage range. Among such converters can be highlighted reconfigurable DAB^52^, reconfigurable T-type DAB^53^, reconfigurable LLC^54^ converters. Multilevel converters due to several levels of output voltage also may extend voltage variation. In practice multilevel DAB chargers are often designed based on Neutral point clamped (NPC) DAB^55^, CLLC NPC DAB^56^, Three-phase Triple-Voltage LLC (T2-LLC)^57^. Figure 11*b*,* c*,* f* show the analyzed reconfigurable topologies and Fig. 13 *a*, *d*, *e* multilevel topologies with their cost.

As shown in Fig. 11 the single-stage charger topologies has additional transistors that increase their cost. More convenient way for providing a wide voltage range is the use of two-stage topologies. As usual, in such two-stage converter first stage is based on common DAB converter whereas second stage may be designed based on some bidirectional DC-DC converter, among them are half-bridge^58^, Cuk^59^, SEPIC / Luo^59^, interleaved half-bridge^60^, three level flying capacitor^61^, three level buck^62^ converters. For such charger topology each stage regulate voltage only in double range that is more realistic. Converters of each stage of two-stage charger are shown in Fig. 12.

As result of comparing of single- and two-stage charger cost it is obviously that wide voltage range regulation with a single-stage converter is very expensive because it contains a high number of transistor, therefore minimum cost of single-stage charger is much higher than in two-stage converter with combination of DAB and half-bridge / parallel three level buck.

After substitution derived charger costs in (16) and (20), ROI of charging stations service with and without stationary energy storage is estimated. If further analysis of the charging service ROI_*ch*_ such assumptions are declared:

- one charger converter cost *C*_*ch*(1)_^*^ is defined by best topology from presented on Fig. 12;
- charging equipment cost *C*_ch_^*^ without stationary battery is *C*_ch_^*^ = *C*_*ch*(1)_^*^ *N*_*ch*_, with stationary battery is *C*_ch_^*^ = (*C*_*ch*(1)_ + 2)^*^N_*ch*_ + *C*_bat_^*^, where sum and 2 is take to account two extra switches as shown in Fig. 12*b*;
- charging equipment maintenance cost *C*_*a*(*ch*)_^*^ is equal to charging equipment cost *C*_ch_^*^ divide by lifetime, *C*_*a*(*ch*)_^*^ = *C*_ch_^*^/*T*_*lf*_;
- night tariff of electricity cost *С*_*e*(*n*)_^*^ is twice as cheap, *С*_*e*(*n*)_^*^ = *С*_e_^*^/ 2 and valid from 11 p.m. to 7 a.m.;
- reactive power amount *Q*_Σ_ is 5% of the substation nominal power *P*_*TS*(*nom*)_, *Q*_Σ_ = 0.05 *P*_*TS*(*nom*)_;
- cosφ penalty level is 0.7, cosφ > 0.7;
- penalty fees *С*_p_^*^ is equal to electricity cost *С*_e_^*^, *С*_p_^*^ = *С*_e_^*^.

Taking to account of the mentioned assumptions the initial data for the charging station design and ROI_*ch*_ calculation are listed in Table 7.

**Table 7:** Initial data for the charging station design and ROI_*ch*_ calculation.

Parameter	Value
Reactive power *Q*_Σ_	67 kVAR
Minimum substation active power *P*_*TS*(min)_	67 kW
Minimum EV consumption power *P*_*EV*(min)_	8 kW
Number of chargers *N*_*ch*_	9
Idle mode probability *p*_*id*_	0.48
Idle mode probability at night *p*_*id*(*n*)_	0.31
Capacity factor CF_*ch*_	0.032
Battery capacity *Cp*_*bat*(1)_	47 A·h
Average electricity cost *C*_*e*(*av*)_^*^	0.68*C*_e_^*^
Charger voltage range coefficient *k*_*ch*_	4

The results of ROI_*ch*_ calculation for both types of charging stations are shown in Fig. 13 in dimensions of difference charging and electricity cost coefficient *k*_*s*(*ch*)_ and level of the EV charging service use *k*_*c*_. Due to less equipment cost, charging service without the stationary battery has large profitability and potentially has income up to 50% with 9–10 times difference in cost of the charging service and electricity cost. But, obviously, such high difference lowered demand of the service that reflected on reducing service use coefficient *k*_*c*_. More realistic level of ROI in 10–20% is achieved with a competitive service cost that exceeds the cost of electricity only by 3–4 times. The charging service with the stationary battery has lower profitability because of use of additional equipment. To compensate that and decrease installation expenses, cheaper or pre-owned batteries may be used. As result, the EV charging service allows to bring a stable income with a rate of 10–20%.

### Modernization stage 2

Typical traction substation with three-phase bridge rectifiers has typical input current distortion with THD_*s*_ = 0.3 and with multiphase topology THD_*mp*_ = 0.12. Even without reactive power generation, with such THD values upper limit, power factor is *PF*_*s*(max)_ = 0.958 and *PF*_*mp*(max)_ = 0.993. Thus, taking into account the generation of reactive power, *PF* penalties may be applied when the substation power factor *PF*_*s*_ is lower than required value *PF*_min_. The minimum value of power factor is achieved with minimum active power *P*_*TS*(min)_, therefore penalty is eliminated when:

26 $$P{F_s}=\frac{{{P_{ST(\hbox{min} )}}}}{{\sqrt {\left( {{P_{ST(\hbox{min} )}}^{2}+Q_{\Sigma }^{2}} \right)\left( {1+{\mathrm{TH}}{{\mathrm{D}}_s}^{2}} \right)} }}{\mathrm{>}}P{F_{\hbox{min} }}{\mathrm{.}}$$

Profit of improving current shape with the active filter *C*_*pr_lf*(*AF*)_^*^ is depended on penalties:

27 $${C_{pr\_lf(AF)}}^{*}={Q_\Sigma }{T_{lf}}\left( {{C_p}^{*}\left( {P{F_{(\hbox{min} )}} - P{F_s}} \right)} \right)={P_{AF}}{C_p}^{*}{T_{lf}}.$$

Value of ROI_*AF*_ also defined by active filter cost *C*_AF_^*^, its maintenance *C*_*a*(*AF*)_^*^ and the active filter efficiency η_*AF*_ that defines of consuming electricity amount:

28 $${\mathrm{ROI}}_{AF} = \frac{{C_{pr\_lf(AF)}^{*} - (C_{AF}^{*} + T_{lf} (C_{a(AF)}^{*} + (1 - \eta_{AF} )C_{e}^{*} P_{AF} ))}}{{T_{lf} (C_{AF}^{*} + T_{lf} (C_{a(AF)}^{*} + (1 - \eta_{AF} )C_{e}^{*} P_{AF} ))}} \cdot 100\% .$$

**Fig. 14:**
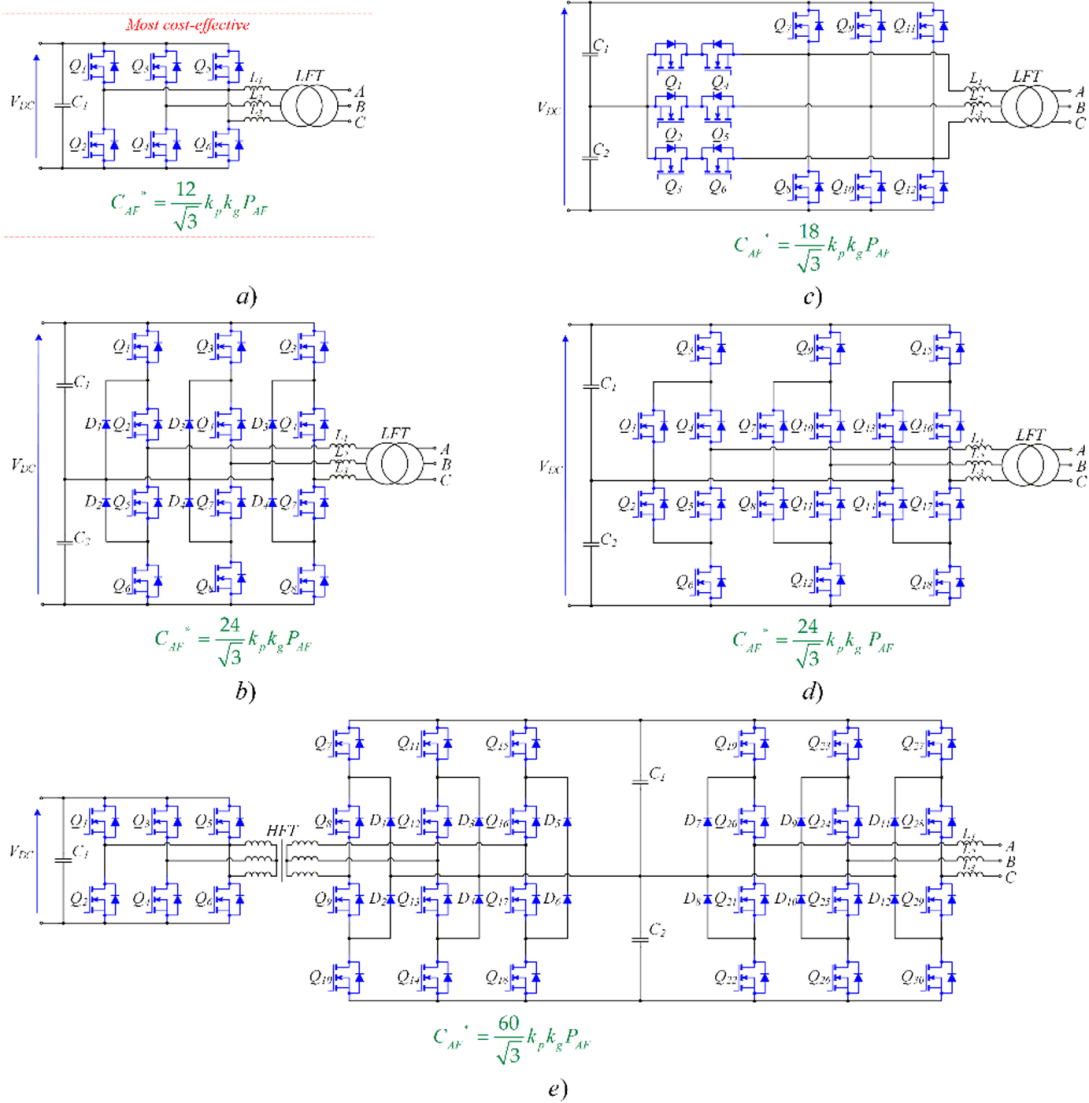
Active filter topologies: (**a**) three-phase active bridge; (**b**) three-phase NPC active bridge; (**c**) three-phase four-wire T-type inverter with neutral inductor; (**d**) fully active NPC topology; (**e**) high-frequency topology with three-phase active bridge.

The active power filter may be designed based on power converters with wide range of topologies (Fig. 14). Most of them are based on low frequency transformer, among them a basic topology with three-phase active bridge^63^, three-phase NPC active bridge^64^ that used for reducing output filter and transistor operating voltage, fully active NPC topology that allows reduce power loss^65^, three-phase four-wire T-type inverter with neutral inductor with reduced number of transistors^66^, Nowadays, for reducing an active filter volume also topologies with high-frequency transformer are used^67^.

**Fig. 15:**
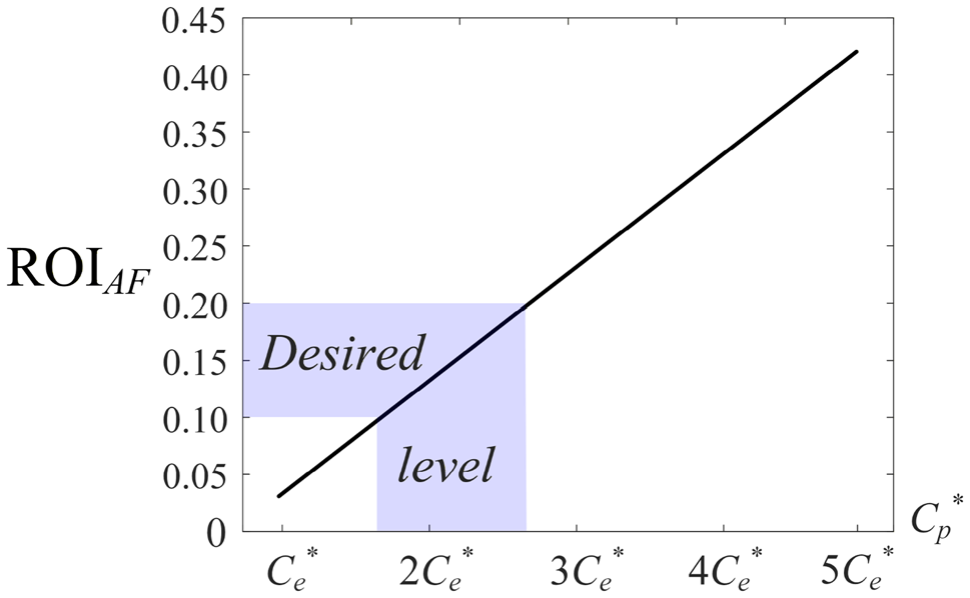
Value of ROI_*AF*_ depend on penalty fees *C*_p_^*^

The active filtering service profitability may be estimated after further assumptions: (1) the active filter maintenance cost *C*_*a*(*AF*)_^*^ is equal to charging equipment cost *C*_AF_^*^ divide by lifetime, *C*_*a*(*AF*)_^*^ = *C*_AF_^*^/*T*_*lf*_; (2) the active filter efficiency is η_*AF*_ = 0.9; (3) topology with common three-phase bridge topology has lowest cost due to a minimum number of transistors. As a result, value of ROI_*AF*_ depend on penalty fees *C*_p_^*^ is shown in Fig. 15.

According analysis ROI_*AF*_ behavior on Fig. 15, once may concluded that for achieving desirable ROI_*AF*_ in range 10–20%, penalty fees should exceed electricity cost in 1.5–2.7 times. For conventional penalty fees that equal electricity cost (*C*_p_^*^ = *C*_e_^*^), such service with proposed assumptions operates with ROI_*AF*_ = 3%.

### Modernization stage 3

Modernization stage 3 aimed for integration of PV based RES. An integral part of this solution is the maximum power point tracker (MPPT) that is built based on power converter^68^. MPPT topology and components of RES system is defined its type, i.e. standalone or grid-connected^69^. In standalone system MPPT topology depends on ratio of input and output voltage range may be built based on conventional buck, boost or buck-boost converter^70^. A generated energy is accumulated on a battery and may be connected to stationary storage system of EVs charger. Such connection allows to reduce consumption from the grid during the day when electricity is more expensive. The grid-connected RES system generates energy directly to AC grid that may be implemented via additional inverter. In the case of a feed-in tariff, the grid-connected solution has more profit, therefore further analysis is focused on it. RES expenses are defined by RES *C*_RES_^*^ and inverter *C*_inv_^*^ cost as well as their maintenance costs *C*_*a*(*RES*)_^*^ and *C*_*a*(*inv*)_^*^ respectively. Since the ROI_*PV*_ does not depend on the power of the installed RES, the system is analyzed from the normalized power with parameters *C*_*RES*(1)_^*^, *C*_*inv*(1)_^*^, *C*_*a*(*RES*)(1)_^*^, *C*_*a*(*inv*)(1)_^*^ respectively. The main factor of ROI_*PV*_ level is the rate of the feed-in tariff, the value of which is determined by the factor *k*_*FN*_ as shown below:

29 $${\mathrm{RO}}{{\mathrm{I}}_{PV}} = \frac{{{T_{lf}}{k_{FN}}{C_e}^* - ({C_{RES(1)}}^* + {C_{inv(1)}}^* + {T_{lf}}({C_{a(RES)(1)}}^* + {C_{a(inv)(1)}}^* + {C_e}^*))}}{{{T_{lf}}({C_{RES(1)}}^* + {C_{inv(1)}}^* + {T_{lf}}({C_{a(RES)(1)}}^* + {C_{a(inv)(1)}}^* + {C_e}^*))}} \cdot 100\% .$$

At the same time, the cost of RES based on a solar battery with normalized power *C*_*RES*(1)_^*^ as well as its maintenance cost *C*_*a*(*RES*)(1)_^*^ are determined by its nominal power *P*_*RES*(1)_ = 1 W:

30 $${C_{RES(1)}}^{*}={T_{lf}}{C_{a(RES)(1)}}^{*}={k_p}{P_{RES(1)}}={k_p}.$$

**Fig. 16:**
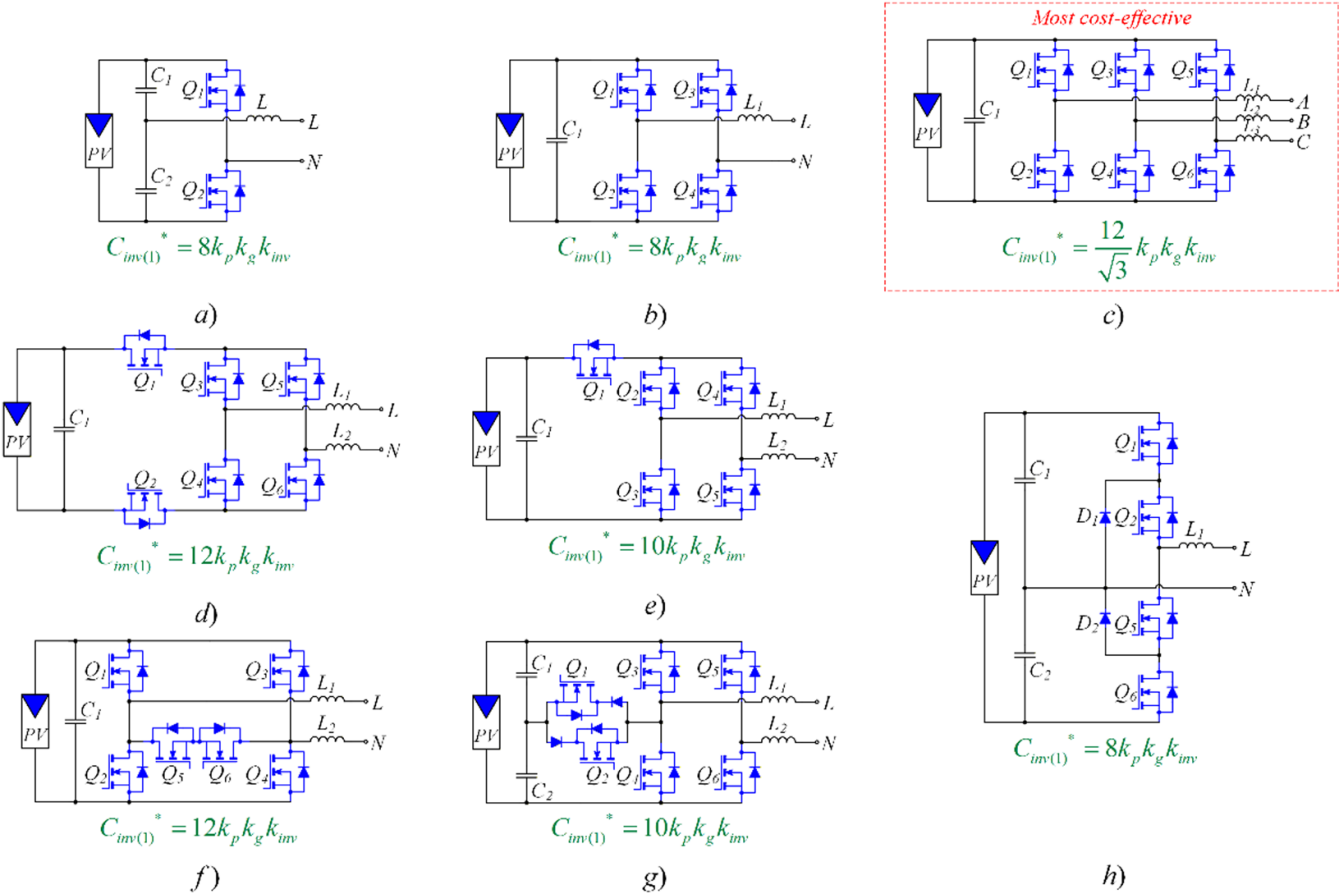
Typical topologies of grid-connected RES systems and their cost: (**a**) half-bridge; (**b**) full-bridge; (**c**) three phase full-bridge; (**d**) H6; (**e**) H5; (**f**) HERIC; (**g**) 3 L-SC; (**h**) 3 L-NPC.

The inverter cost depends of its topology. As usual they are implemented on bridge converters with hard or soft switching modulation^71^(Fig. 16). Non-isolated single-phase inverters suffer from leakage current that flows between AC and RES system^72^. Improved more complex HERIC type topologies treat the disadvantage^73^. Transistor operating voltage reducing is achieved in multilevel inverters with cascaded H-bridge^74^, 3 L-NPC^75^ or 3 L-SC^76^ topologies. For three phase systems, as usual, common three phase bridge inverters provide inexpensive and effective MPPT solutions^77^.

Except power converter topology its cost also depends on input and output voltage/current variation range. Maximum power point voltage *U*_*MPP*_ of solar battery, as usual, is in range 72–76% of its open circuit value *U*_*OC*_, *U*_*MPP*_ = 0.72.0.76 *U*_*OC*_, whereas maximum power point current *I*_*MPP*_ lays in range 90–93% of short circuit current *I*_*SC*_, *I*_*MPP*_ = 0.9.0.93 *I*_*SC*_^70^. Therefore, factor of increasing cost of inverter because of voltage and current variation *k*_*inv*_ is:

31 $${k_{inv}}=\frac{{{U_{OC}}{I_{SC}}}}{{{U_{MPP(\hbox{min} )}}{I_{MPP(\hbox{min} )}}}}=1.54.$$

**Fig. 17:**
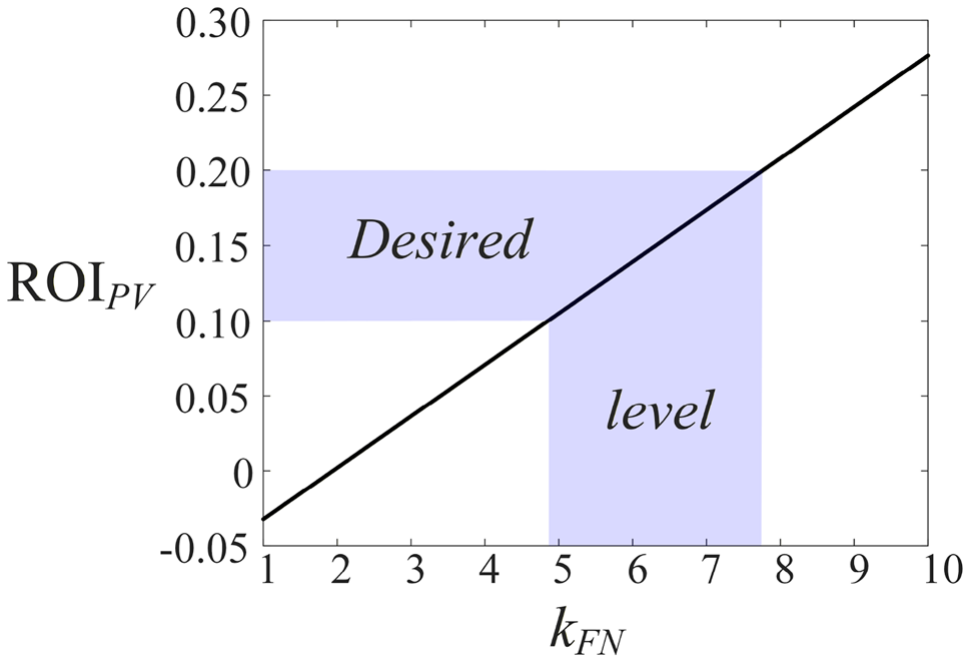
ROI_*PV*_ as function of feed-in tariff factor *k*_*FN*_.

If the inverter maintenance *C*_*a*(i*nv*)(1)_^*^ is determined via its power cost as follows *C*_*a*(i*nv*)(1)_^*^ = *C*_i*nv*(1)_^*^ / *T*_*lf*_, value of ROI_*PV*_ may be defined as function of feed-in tariff factor *k*_*FN*_ as shown in Fig. 17. As shown in Fig. 17 for achieving desired ROI_*PV*_ level 10–20%, feed-in tariff has to exceed electricity cost 5–8 times, which is usual value for most countries^78,79^.

**Fig. 18:**
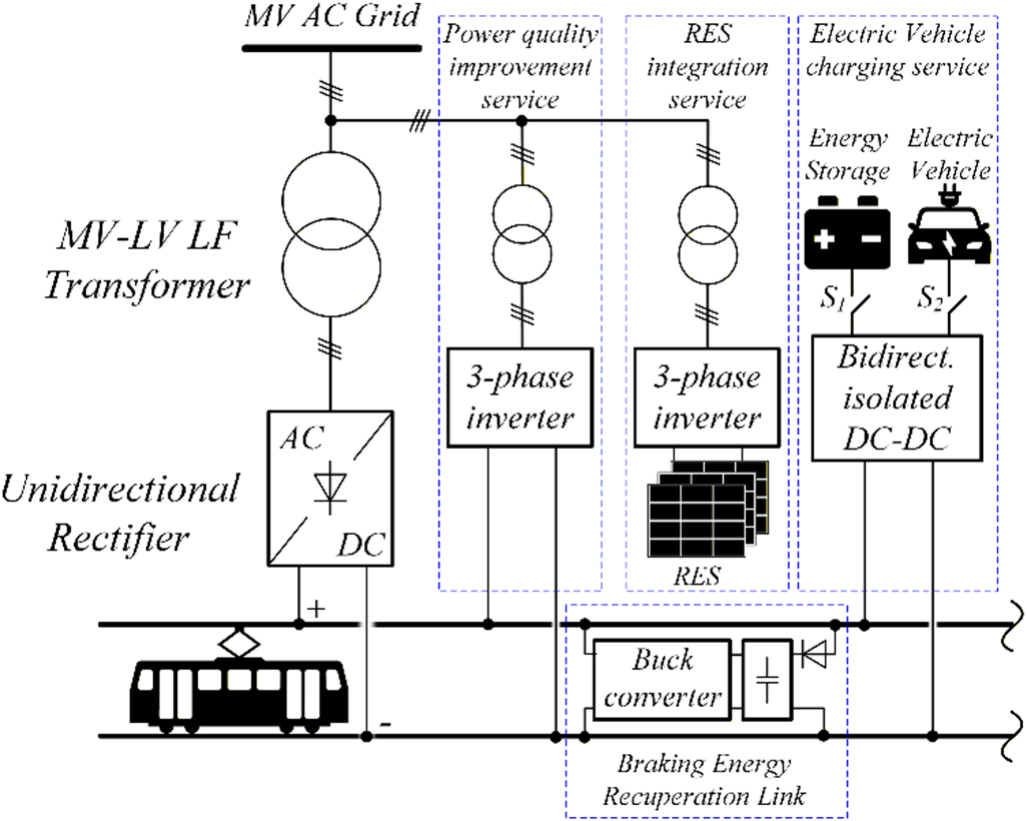
Proposed structure of a power converter interface for improving the efficiency and profitability of the traction DC grid.

According to previous analysis the power converter interface after all modernization stages has the topology shown in Fig. 18.

The designed PCI enables the following objectives to be achieved:

- integration of electric vehicle charging infrastructure into DC traction grids, while mitigating grid stress caused by charging loads;
- elimination of penalties associated with non-compliant power factor (PF) and reactive power (cosφ) values;
- integration of renewable energy sources (RES) into the traction grid power supply system.

## Conclusions

The increase of electric vehicles number necessitates the modernization of traction grids, which can help to reduce power grid overloads, improve power quality and enable the integration of renewable energy sources. As usual, modernization requires substantial investment in equipment renovation and should be economically beneficial for the grid owner. This paper focuses on the conditions that support the gradual replacement of outdated equipment with new systems, designed to balance EV consumption from the grid and improve power quality. Using the example of a DC traction substation, the study derives conditions, based on standard cost models for power converter interfaces, electric batteries and renewable energy sources, that make it possible to implement EV charging, power quality improvements and renewable integration with a return on investment (ROI) of 10–20%.

To increase profitability, the article proposes a phased modernization of substations. Each subsequent stage is implemented when favorable conditions for modernization arise. Stage 0, which is related to eliminating overvoltage during regenerative braking, is the least costly and can be implemented under any conditions, providing an additional ~ 2% increase in profit. The stage 1 provides penalties mitigation during traction substation idle operation via implementing charging service. The stage implementation becomes profitable with a competitive service cost that exceeds the cost of electricity at least by 3–4 times. Stage 2 enhances the quality of the current drawn from the power grid and becomes economically viable when penalty fees exceed electricity costs by a factor of 1.5–2.7. The last one modernization stage integrates PV-array to traction grid when feed-in tariff has to exceed electricity cost 5–8 times. The obtained ratios indicate, that under current levels of penalties, electricity prices and feed-in tariffs, the costs of modernization can potentially be offset by the high profitability of these new services, including charging, power quality improvement and renewable integration, highlighting an opportunity to significantly reduce dependence on fossil fuels and lower CO₂ emissions in the cities.

It is important to note, that the present study has several limitations that should be considered when interpreting the results. The analysis is based on simplified system-level models and representative parameter values, which do not fully capture dynamic system behavior, time-varying interactions between subsystems or differences in regulatory environments. In addition, the proposed framework focuses on economic feasibility and does not explicitly address control complexity or stability aspects of multiport converter operation. Future work will therefore focus on detailed simulation-based validation under realistic load conditions, sensitivity analysis with respect to tariffs and penalty structures, and the development of advanced control strategies to support reliable operation of integrated traction energy systems.

## Data Availability

All data generated or analysed during this study are included in this published article.
